# A Histone Deacetylase Adjusts Transcription Kinetics at Coding Sequences during *Candida albicans* Morphogenesis

**DOI:** 10.1371/journal.pgen.1003118

**Published:** 2012-12-06

**Authors:** Denes Hnisz, Anaïs F. Bardet, Clarissa J. Nobile, Andriy Petryshyn, Walter Glaser, Ulrike Schöck, Alexander Stark, Karl Kuchler

**Affiliations:** 1 Medical University Vienna, Christian Doppler Laboratory for Infection Biology, Max F. Perutz Laboratories, Vienna, Austria; 2 Institute of Molecular Pathology (IMP), Vienna, Austria; 3 Department of Microbiology and Immunology, University of California San Francisco, San Francisco, California, United States of America; 4 GATC Biotech AG, Konstanz, Germany; University of California San Francisco, United States of America

## Abstract

Despite their classical role as transcriptional repressors, several histone deacetylases, including the baker's yeast Set3/Hos2 complex (Set3C), facilitate gene expression. In the dimorphic human pathogen *Candida albicans*, the homologue of the Set3C inhibits the yeast-to-filament transition, but the precise molecular details of this function have remained elusive. Here, we use a combination of ChIP–Seq and RNA–Seq to show that the Set3C acts as a transcriptional co-factor of metabolic and morphogenesis-related genes in *C. albicans*. Binding of the Set3C correlates with gene expression during fungal morphogenesis; yet, surprisingly, deletion of *SET3* leaves the steady-state expression level of most genes unchanged, both during exponential yeast-phase growth and during the yeast-filament transition. Fine temporal resolution of transcription in cells undergoing this transition revealed that the Set3C modulates transient expression changes of key morphogenesis-related genes. These include a transcription factor cluster comprising of *NRG1*, *EFG1*, *BRG1*, and *TEC1*, which form a regulatory circuit controlling hyphal differentiation. Set3C appears to restrict the factors by modulating their transcription kinetics, and the hyperfilamentous phenotype of *SET3*-deficient cells can be reverted by mutating the circuit factors. These results indicate that the chromatin status at coding regions represents a dynamic platform influencing transcription kinetics. Moreover, we suggest that transcription at the coding sequence can be transiently decoupled from potentially conflicting promoter information in dynamic environments.

## Author Summary

Many human pathogenic fungi are able to change their morphological properties, including their size and shape, in response to their outside environment. This ability, which is key for infection, is not completely understood on the molecular level. We have previously shown that not just DNA–binding transcription factors, but also chromatin-modifying enzymes that interact with DNA–binding proteins, are important regulators of morphogenesis in the model fungus *C. albicans*. In this work we dissect how such a chromatin-modifying enzyme regulates fungal morphogenesis. We surprisingly found that perturbation of chromatin has little influence on steady-state transcription, but modulates transient gene expression changes in differentiating *C. albicans* cells. Altered transcription kinetics affects a group of transcription factor genes that determine morphology. We thus identified a chromatin modifier that exerts kinetic control of transcription factor genes to control fungal morphogenesis. The results highlight the importance of chromatin to determine the kinetics of transcription changes rather than the steady-state transcript levels.

## Introduction

Cells with identical genomes can adopt various phenotypes, which is a central feature during differentiation of multicellular organisms. During differentiation, the outside stimuli and the cellular machineries that process them are thought to determine the resulting cell types. Often, both the original and the resulting cell types are stable and display defined morphologies and gene expression programs. Understanding how such transitions between two stable cellular states are controlled and achieved on the molecular level is of central importance for understanding development and the relationship of organisms with their environment.

Unicellular species such as simple fungi can also undergo differentiation. For instance, pleiomorphic fungi, such as the opportunistic human pathogen *Candida albicans* display diverse morphologies ranging from unicellular yeast-like, to multicellular pseudohyphal, and hyphal structures [1], [2]. The ability to undergo reversible transitions between the distinct morphologies is a key virulence factor of *C. albicans*, shared by many other pathogenic fungi of even distantly related taxa such as *Histoplasma* and *Cryptococcus* [2], [3]. Consequently, blocking fungal morphogenesis represents a plausible antifungal therapeutic strategy [4].

Hyphal differentiation of *C. albicans* is responsive to environmental and host stimuli and is controlled by several signal transduction cascades and over thirty transcriptional regulators [5], [6], [7], [8]. Nevertheless, how the individual factors interact and how they integrate information from upstream signaling cascades is poorly understood. Recently, several studies have implicated chromatin and chromatin-modifying enzymes in the signal integration process. For example, the NuA4 histone acetyltransferase (HAT) and the Hda1 histone deacetylase (HDAC) mediate dynamic acetylation and deacetylation of histones at promoter regions of hypha-specific genes, and their proper function is required for the establishment of a normal filamentation expression program [9]. In yeast-phase cells, hyphal-specific genes are repressed by the transcription factor Nrg1 [10], [11]. During hyphal initiation, cyclic adenosine monophosphate (cAMP)/protein kinase A (PKA) signaling drives eviction of Nrg1 from its target promoters, where Hda1 is recruited by another transcription factor, Brg1 [10], [12]. Hda1 activity subsequently results in the eviction of NuA4 from the target promoters, which prevents Nrg1 rebinding [10]. In this model, promoter chromatin is perceived as a platform for temporally regulated transcription changes in morphogenesis. Consistent with this notion, several other chromatin modifier mutants, including the histone methyltransferase Set1 [13], the HAT Rtt109 [14], and the Set3 HDAC complex [15] display morphogenesis-related phenotypes.

The Set3 Complex (Set3C) was first identified as a repressor of sporulation in *Saccharomyces cerevisiae*, and sequence homology suggests that it is evolutionarily conserved from fungi to mammals [16]. The catalytic subunit Hos2 was the first identified non-canonical HDAC required for gene activity [17]. In *S. cerevisiae*, the Set3C occupies the coding sequence of highly transcribed genes and is required for full expression of the galactose-inducible gene cluster [17]. Deacetylation of nucleosomes within the coding regions is thought to reset chromatin to a permissive state facilitating repeated cycles of transcription [17], [18]. Homologues of the Set3C in other fungal species have been implicated in the regulation of morphogenesis and virulence [19], [20]. We have recently shown that the *C. albicans* Set3C acts as a repressor of hyphal differentiation and its function requires functional cAMP/PKA signaling [15].

In this study, we set out to identify the molecular mechanism through which the *C. albicans* Set3C controls morphogenesis. First, we used a combination of chromatin immunoprecipitation followed by sequencing (ChIP-Seq) and RNA sequencing (RNA-Seq) to define the genome-wide regulatory target genes of the Set3C in yeast and hyphal cells. We found that similar to its *S. cerevisiae* homologue, the CaSet3C exclusively decorates coding regions and is associated with high transcriptional activity. However, during transient hyphal-inducing conditions, the Set3C delays the establishment of the hyphal-specific gene program by modulating the transcript levels of four phase-specific transcription factors (*BRG1*, *TEC1*, *NRG1*, *EFG1*), suggesting that the Set3C can act both as a transcriptional activator and repressor. We also demonstrate that these four factors form a core transcriptional circuit underlying morphogenesis, whose output is restricted by the Set3C. The results provide comprehensive insights into the mechanisms whereby the chromatin layer of regulation superimposes on a core transcriptional factor circuit that controls cellular morphogenesis in *C. albicans* and possibly in other fungal pathogens.

## Results

### The *C. albicans* Set3C is a coding sequence histone deacetylase

**Figure 1:**
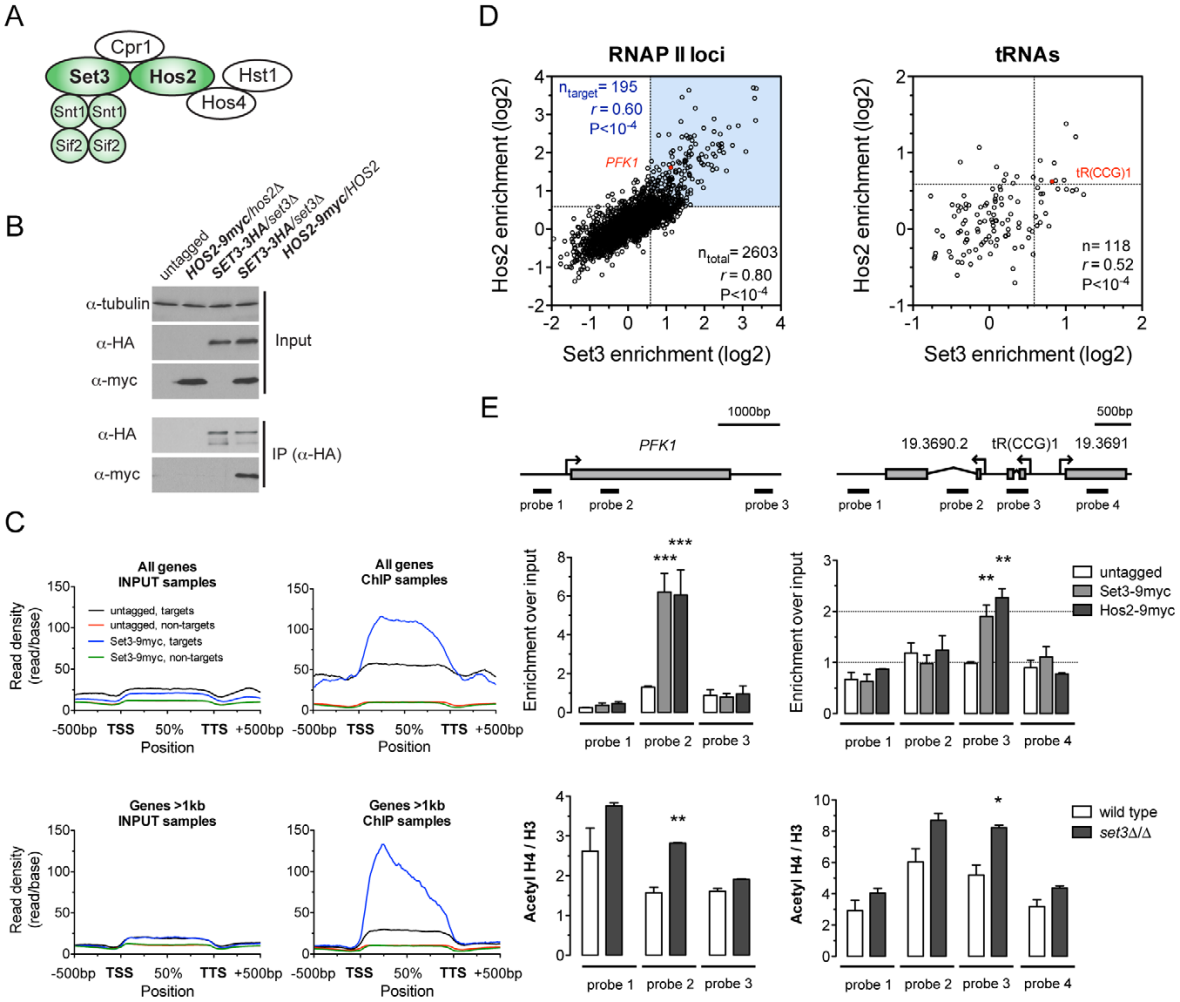
The *C. albicans* Set3C is a coding sequence histone deacetylase. (A) Architecture of the *S. cerevisiae* Set3C. The subunits among which physical interaction was confirmed in *C. albicans* are colored green (see Figure S1B). (B) Physical interaction of Set3 and Hos2. Set3-3HA was immunoprecipitated from whole cell extracts and the interaction was probed by immunoblot detection of a Hos2-9myc allele. (C) Read density profiles of one replicate of a Set3-9myc and an untagged control ChIP-Seq experiment. Genes were divided into binding targets (“targets”) and non-targets (see Materials and Methods). Transcription start site (TSS) denotes the start codon and Transcription termination site (TTS) denotes the stop codon. The read density values between the TSS and TTS were calculated to a percentage scale, and 500 bases upstream of the TSS and downstream of the TTS were included. On the bottom panel only genes with a coding region longer than 1 kilobase were included. (D) Definition of CaSet3C refined target gene set. Each dot corresponds to one ORF. Binding targets of RNAPII transcribed genes are defined as having an at least 2-fold enrichment on one axis and an at least 1.5-fold on the other axis (blue box). Target tRNA loci are defined as having an at least 1.5-fold enrichment on both axes. The complete dataset is found in Tables S4 and S5. “*r*” denotes a Pearson's correlation coefficient. (E) The Set3C functions as histone deacetylase *in vivo*. Top panel: validation of Set3 and Hos2 binding using the indicated probes around the *PFK1* and *tR(CCG)1* loci by qPCR. Values are normalized to a fragment of the *ADE2* locus. Bottom panel: ChIP experiments were performed with antibodies against acetylated histone H4 and the C-terminus of histone H3. The qPCR values at the probe positions were normalized to a fragment of the telomere of Chromosome 7. The ratio of the signal of the acetylated H4 ChIP and H3 ChIP is shown on the y-axis. Data are shown as mean+SD of three independent experiments. Statistical significance was determined by two-tailed t-test relative to the control values. *P<0.05, **P<0.01, ***P<0.001.

In *S. cerevisiae*, the Set3C is composed of seven different subunits, which show sequence conservation in *C. albicans* and mammals [15], [16]. In *S. cerevisiae*, four of the subunits form a core complex (Set3, Hos2, Snt1, Sif2) and are required for structural integrity, while three additional subunits are peripheral (Hst1, Cpr1, Hos4) [16]. To enable biochemical investigation of the CaSet3C, we constructed a series of *C. albicans* strains carrying epitope-tagged alleles of the Set3 and Hos2 subunits. *C. albicans* is diploid, thus the second alleles in these strains were deleted. Since deletion of the Set3C causes hyperfilamentation [15], phenotypic analysis ensured that the tagged alleles were functional (Figure S1A). To probe the conservation of the complex architecture, we immunoprecipitated Set3 and Hos2 from whole cell extracts, and identified their interaction partners by mass spectrometry. Both subunits co-purified with the homologues of all *S. cerevisiae* core complex subunits (Set3, Hos2, Snt1, Sif2) (Figure S1B), indicating that the core complex is conserved in *C. albicans* (Figure 1A). In addition, we verified the Set3-Hos2 interaction by immunoprecipitation and immunoblotting (Figure 1B) and confirmed nuclear localization of the complex with a Hos2-GFP construct (Figure S1C).

To obtain a genome-wide binding profile of the Set3C and to determine its regulatory targets, we performed chromatin immunoprecipitation followed by sequencing (ChIP-Seq) of Set3 and Hos2 in exponentially growing yeast-phase cultures. We first identified binding peaks using Model-based Analysis of ChIP-Seq (MACS) [21], and found that 90% of all peak summits and around 85% of positions within peak regions fall within annotated coding regions (Figure S3A). Read density profiles averaged across all genes (*meta-gene analyses*) confirmed the strict localization of binding to coding regions (Figure 1C), and suggested that binding of Set3 and Hos2 to each gene across conditions could be assessed quantitatively by read-coverage of the coding regions (in reads-per-kilobase-per-million-reads [RPKM]) similar to RNA-Seq analyses; see Materials and Methods). Set3 and Hos2 enrichments showed a strong correlation genome-wide both for RNA Polymerase II (RNAPII)-transcribed genes (*r* = 0.80, Pearson's correlation) and RNAPIII-transcribed tRNA loci (*r* = 0.52, Pearson's correlation), arguing that both subunits co-localize on the chromosome (Figure 1D).

To test whether the Set3C indeed functions as a histone deacetylase *in vivo*, we performed ChIP experiments of acetylated histone H4. The ratio of acetylated H4 to total histone H3 was increased in a *set3*Δ/Δ strain when compared to wild type at the Set3C-bound positions (Figure 1E). Taken together, these results demonstrate that the *C. albicans* Set3C has histone deacetylase activity and localizes to coding regions of its target genes and tRNA loci.

### The Set3C decorates highly transcribed genes

**Figure 2:**
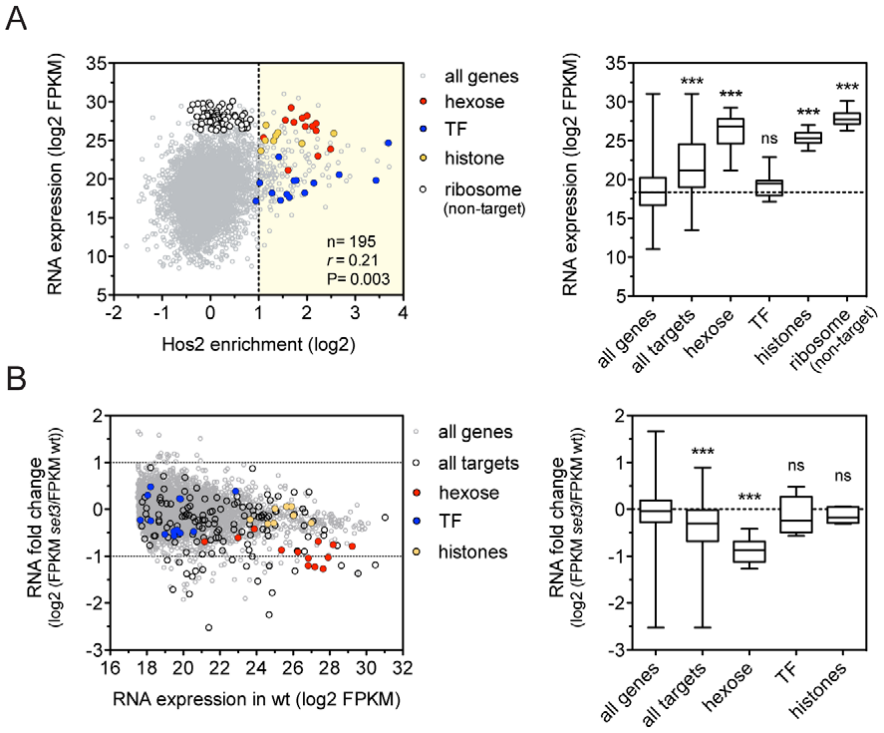
The Set3C decorates highly transcribed genes. (A) Correlation of Set binding and RNA expression for the target gene set (defined on Figure 1D). Each dot corresponds to one gene. The distribution of expression values of the genes belonging to each functional category is shown on the right panel. “*r*” denotes a Pearson's correlation coefficient. TF stands for the transcription factor cluster (see text). Statistical significance was determined by the Mann-Whitney U-test relative to the “all genes” set. *P<0.05, **P<0.01, ***P<0.001, ns: not significant. (B) Transcript profile of *set3*Δ/Δ cells by RNA-Seq. The fold change in RNA expression between *set3*Δ/Δ and wild type cells at each gene is plotted against the expression level of the gene in wild type cells. The direct binding targets and their functional groups are highlighted. The distribution of fold changes of the genes belonging to each functional category is shown on the right panel. Statistical significance was determined by the Mann-Whitney U-test relative to the “all genes” set. *P<0.05, **P<0.01, ***P<0.001, ns: not significant.

To dissect how the Set3C regulates gene transcription, we performed RNA-Sequencing (RNA-Seq) of exponentially growing yeast-phase cultures. We found that *C. albicans* Set3C target genes were on average 8.7-fold more highly expressed than all genes (Figure 2A), and that binding correlated with RNA expression, as described for the *S. cerevisiae* homologue (Figure S4A) [17]. In particular, hexose catabolism genes and nucleosomal histone genes (both enriched among Set3C targets; P = 1.3×10^−10^, P = 8×10^−8^, respectively) are both highly occupied and expressed (Figure 2A). This implies that Set3C might be involved in enhancing gene expression of its target genes. However, surprisingly, when we performed RNA-Seq of a *SET3*-deletion mutant, the expression levels of only few target genes were affected, including the hexose catabolic genes (Figure 2B, Figure S4B). In contrast, histone genes and transcription factors, another functional category enriched among the targets (P = 2×10^−3^) remained unaffected. In wild type cells, acetylation level of histone H4 at the coding sequences of selected Set3C-targets correlated with RNA expression (*r* = 0.88, Pearson's correlation, Figure S4D). Moreover, increased acetylation of histone H4 was detectable at almost all tested loci in *set3*Δ/Δ cells (Figure S4C). These data argue that the occupancy of Set3C is linked to active transcription, but the presence of Set3C at transcribed gene bodies influences the steady-state transcript levels of only a small subset of targets.

### Set3C recruitment predicts induction, while depletion predicts repression

**Figure 3:**
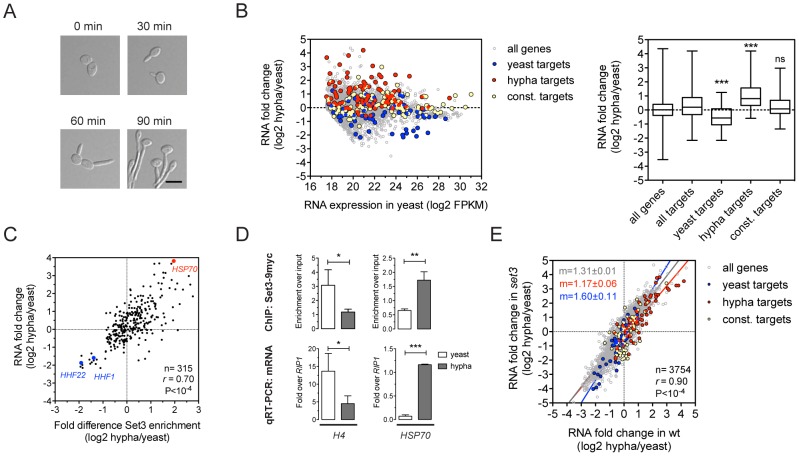
Set3C recruitment predicts induction and depletion predicts repression. (A) Microscopic images of cells undergoing yeast-to-hypha differentiation. The cells at each time point do not correspond to the cells at the other time points. Scale bar corresponds to 5 µm. (B) Transcript landscape of hyphal cells 30 minutes after induction. The fold change in RNA expression between hyphal and yeast cultures at each gene is plotted against the expression level of the gene in wild type yeast cells measured by RNA-Seq. Each dot represents one gene. Set3C binding targets were defined by Set3C ChIP-Seq experiments (see Materials and Methods). The Set3C target genes are divided into yeast-specific, hypha-specific and constitutively bound subgroups. The distribution of RNA fold changes of the genes belonging to each category is shown on the right panel. Statistical significance was determined by the Mann-Whitney U-test relative to the “all targets” set. *P<0.05, **P<0.01, ***P<0.001, ns: not significant. (C) Correlation of RNA fold change and differential ChIP enrichment signals. Each dot corresponds to one gene, and only the genes defined as Set3C binding targets in at least one phase are shown. “*r*” denotes a Pearson's correlation coefficient. (D) qPCR verification of the correlation on (C). Histone H4 has two loci in *C. albicans* (*HHF1* and *HHF22*), and the primers used in the qPCR bind alleles of both. Data are shown as mean+SD of three independent experiments. Statistical significance was determined by two-tailed t-test. *P<0.05, **P<0.01, ***P<0.001. (E) Comparison of the gene induction profiles of wild type and *set3*Δ/Δ cells undergoing hyphal differentiation. Fold change between the hyphal and yeast phases for the two genotypes are plotted on the two axes. Each dot corresponds to one gene. The categories of Set3C binding targets are defined as on (B). “*r*” denotes a Pearson's correlation coefficient, and “m” denotes the slope of the linear regression.

The yeast-to-hypha transition in *C. albicans*, which is repressed by the Set3C [15], involves transcriptional changes that affect around 600 genes, corresponding to roughly 10% of the genome [11], [22]. To further dissect how the Set3C represses this transition, we indentified the binding targets of the Set3C in cells exposed to filament-inducing conditions (Figure 3A). In total, we detected 237 Set3C targets in hyphae, after a 30 minute induction. The ratio of the hypha/yeast ChIP-Seq values was used to classify a hypha-specific target gene set (127), a yeast-specific target set (85) or genes constitutively bound (110) (Table S5). We also performed RNA-Seq during filament formation and found that hypha-specific Set3C targets were on average induced whereas yeast-specific target genes were repressed upon filament-induction (Figure 3B). In fact, the differential RNA expression values and differential ChIP enrichment signals showed a strong correlation both for Set3 (*r* = 0.69, Pearson's correlation) and Hos2 (*r* = 0.8, Pearson's correlation) (Figure 3C, 3D; data not shown). However, when *set3*Δ/Δ cells were induced to form hyphae, their transcript induction profile was virtually identical to that of wild type cells (*r* = 0.9, Pearson's correlation, Figure 3E). These results clearly demonstrate that the Set3C is recruited to induced genes while it is depleted from repressed genes upon hyphal induction. Notably, *set3*Δ/Δ cells are still able to efficiently execute initiation of the hypha-specific transcriptional program.

### The Set3C is a co-factor of glycolysis and morphogenesis regulators

**Figure 4:**
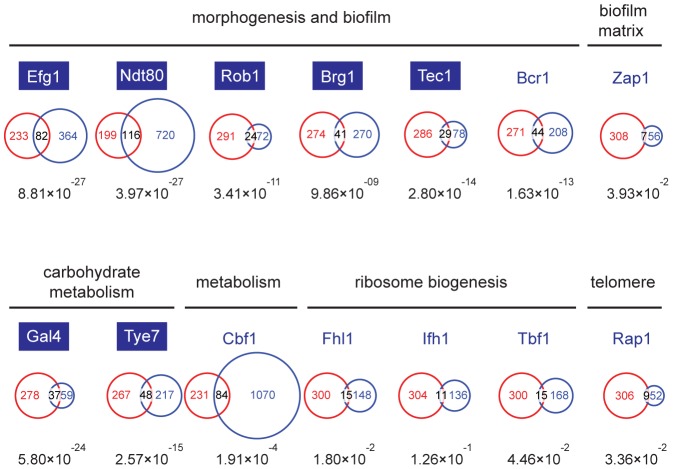
The Set3C is a co-factor of glycolysis and morphogenesis regulators. Statistical analysis of the overlaps of the target genes of selected transcription factors with the target gene set of Set3C in the individual phases. The TF target sets are shown in blue, the Set3C target sets in red, and the TFs are grouped according to the functions they are implicated in. The target sets of the TFs were imported from the following reports: Efg1, Ndt80, Rob1, Brg1, Tec1 and Bcr1 from [23], Zap1 from [24], Gal4 and Tye7 from [25], Cbf1, Fhl1, Ifh1, Tbf1 and Rap1 from [26]. The area of the circles and overlaps are proportional to the number of genes they consist of. For each overlap the P-value of hypergeometric testing is shown. Transcription factors that are themselves binding targets of Set3C in at least one morphological phase are placed in blue boxes.

Though the Set3C appears to be selectively recruited to a subset of transcribed genes, the differences in steady-state transcription and gene induction patterns in *set3*Δ/Δ cells are most surprisingly only minimal, and cannot explain why *set3*Δ/Δ cells are hyperfilamentous. To identify such potentially misregulated loci, we decided to identify which transcription factor(s) are responsible for Set3C recruitment, and collected the target lists of all transcription factors (TFs) whose genome-wide binding has been analyzed in *C. albicans*. These candidate TFs have been implicated in several cellular processes, including morphogenesis and biofilm formation [23], biofilm matrix regulation [24], carbohydrate metabolism [25], ribosome biogenesis [26], telomere control [26] and metabolic pathways [26]. Interestingly, the P-values of the overlaps showed an around 10 order of magnitude difference for TFs involved in morphogenesis (Efg1, Ndt80, Rob1, Brg1, Tec1) and carbohydrate metabolism (Gal4, Tye7) compared to other TFs (Figure 4). The fact that glycolytic genes were enriched in the GO-term analysis (see above) is in agreement with the finding that the Set3C target list shows a significant overlap with the targetome of Gal4 and Tye7, the major activators of the glycolytic gene cluster [25]. To our surprise, we also found that TFs whose target set showed a significant overlap with the Set3C set are themselves Set3C targets (blue boxes, Figure 4). Taken together, these results indicate that Set3C is a transcriptional co-factor of morphogenesis and glycolysis regulators. The fact that the regulators themselves are Set3C targets suggests that misregulation of rather the TF genes and not their targets could be the cause of the hyperfilamentous phenotype displayed by *set3*Δ/Δ cells.

### The Set3C regulates morphogenesis through a transcription factor cluster

**Figure 5:**
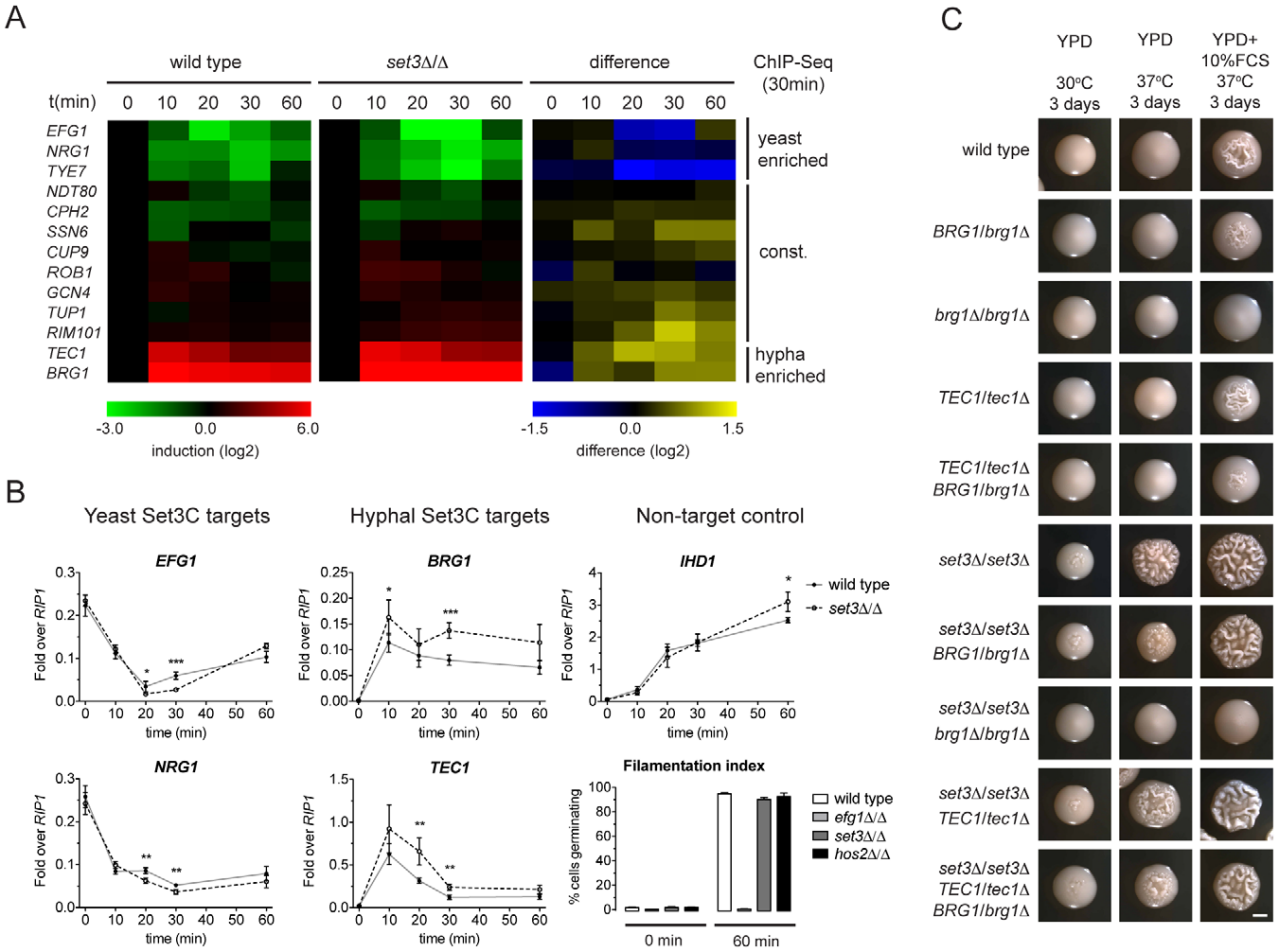
The Set3C modulates morphogenesis through a TF cluster. (A) qRT-PCR quantification of the indicated transcripts in wild type and *set3*Δ/Δ yeast cells induced to differentiate into hyphae 10, 20, 30 and 60 minutes after induction. The transcript levels are normalized against *RIP1* expression and the level of the respective gene in yeast cells (0 min). The right panel shows the quantitative difference between the values in the two genotypes at each individual time point. Average values of four independent experiments are shown. (B) Transcript levels of *BRG1*, *TEC1*, *NRG1* and *EFG1* in differentiating wild type and *set3*Δ/Δ yeast cells as on (A) quantified by qRT-PCR. *IHD1* is hyphal-induced non-Set3C-target control gene. In the bottom right panel the percentage of cells germinating at the starting cultures and 60 minutes post induction is shown as a control. Data are shown as mean+SD of four independent experiments. Statistical significance was determined by two-tailed t-test. *P<0.05, **P<0.01, ***P<0.001. (C) Removal of one *BRG1* allele reverts hyperfilamentation of *set3*Δ/Δ cells under intermediate inducing conditions. Shown are photographs of single colonies. YPD at 30°C supports yeast-phase growth, YPD+FCS at 37°C supports hyphal growth, YPD at 37°C represents “intermediate” conditions. Scale bar corresponds to 2 mm.

While morphogenesis regulators are major Set3C targets, and their target sets show significant overlap with the Set3C targetome, steady-state transcript levels of TFs were unaltered in *set3*Δ/Δ yeast phase cells (Figure 2B). Thus, we hypothesized that if altered transcription of TFs is responsible for the hyperfilamentous phenotype of *set3*Δ/Δ cells, this effect could be transient. Consequently, we analyzed the transcript level changes of the Set3C-target TFs at a high kinetic resolution around the induction stimulus. We found that the transcript levels of *EFG1*, *NRG1*, *TYE7*, which were identified as hyphal-enriched Set3C targets, undergo a rapid decrease following hyphal induction. By contrast, a rapid 50–100-fold induction of transcripts of the hyphal-specific targets *BRG1* and *TEC1* were visible after only 10 minutes (Figure 5A). The TFs bound constitutively by the Set3C did not show significant transcript changes around the induction point. This pattern was qualitatively identical in differentiating *set3*Δ/Δ cells. However, a 1.5–2-fold quantitative difference was observed between wild type and *set3*Δ/Δ cells at several time points that mostly affected the phase-specific TFs (Figure 5A, 5B). For instance, *BRG1* transcript level was about 1.5-fold higher in *set3*Δ/Δ cells at all time points following induction, and *TEC1* transcript level was 2-fold higher in *set3*Δ/Δ cells at 20 and 30 minutes post induction. On the other hand, *EFG1* and *NRG1* transcript levels showed an approximate 1.5–2-fold decrease in *set3*Δ/Δ cells at 20 and 30 minutes, respectively (Figure 5A, 5B). This suggested that the hypha-specific factors reach higher transcript levels in *set3*Δ/Δ cells, while the yeast-specific factors are repressed more upon hyphal differentiation. This effect was not a result of more cells responding to serum in the *set3*Δ/Δ culture, as the non-target *IHD1* gene showed a quantitatively identical induction pattern, and the number of filamenting cells was scored above 90% percent after a 60 minute induction in both genotypes (Figure 5B; additional supporting qRT-PCR data in Figure S5).

If the transient transcript level differences of the phase specific TFs cause the hyperfilamentous phenotype of *set3*Δ/Δ cells, then manipulation of the levels of the TFs should be epistatic to the lack of *SET3*. Indeed, we found that removal of one *BRG1* allele almost completely reverted the hyperfilamentous phenotype of *set3*Δ/Δ cells under intermediate conditions, while deletion of one *TEC1* allele did not (Figure 5C), which is probably explained by the facts that *TEC1* expression shows a burst upon induction as opposed to *BRG1* that stays stably high. These data demonstrate that the Set3C adjusts transient expression of phase-specific morphogenesis regulators during hyphal differentiation, and that genetic interference with the *BRG1* regulator is sufficient to partially revert Set3C-deficiency.

### The four phase-specific Set3C-target TFs form a core transcriptional circuit

**Figure 6:**
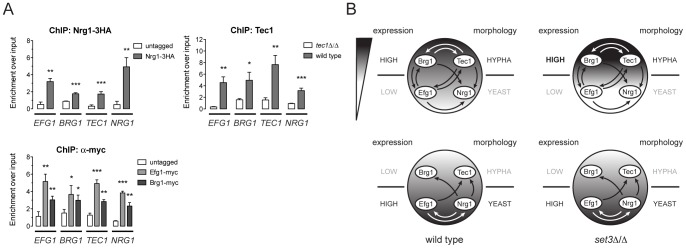
Four phase-specific Set3C-target TFs form a core circuit. (A) qRT-PCR analysis of Nrg1, Tec1, Efg1 and Brg1 binding at the *EFG1*, *BRG1*, *TEC1* and *NRG1* promoters. ChIP experiments were performed in yeast phase for Nrg1 and hyphal phase for Tec1, Efg1 and Brg1. Data are shown as mean+SD of three independent experiments. Statistical significance was determined by two-tailed t-test. *P<0.05, **P<0.01, ***P<0.001. (B) Simplified model of the transcription circuit overlayed with a chromatin pathway to regulate *C. albicans* morphogenesis. The big circle represents the transcript profiles of the four regulators with the indicated scaling. The arrows representing regulatory information are derived from the ChIP experiments at (A). The arrows of Efg1 in the yeast phase are taken from the genome-wide binding data in [27]. The arrows representing autoregulation of each four regulators are omitted for simplicity. Note that in *set3*Δ/Δ cells, the architecture of the TF-circuit remains intact, yet the temporary differences in the regulator mRNA levels are represented by the differences in the grayscale.

Biofilm formation in *C. albicans* is controlled by a transcriptional circuit comprising of six core regulators, including *BCR1*, *BRG1*, *NDT80*, *EFG1*, *ROB1* and *TEC1* [23]. Strikingly, five out of the six factors (all except *BCR1*) are Set3C targets, and three of the factors (*BRG1*, *EFG1* and *TEC1*) showed altered transcription kinetics in *set3*Δ/Δ cells (Figure 5A). We therefore tested if the four phase-specific regulators that display Set3C-dependent transcription kinetics (*BRG1*, *EFG1*, *TEC1* and *NRG1*) also form a regulatory circuit. Remarkably, ChIP experiments revealed that Nrg1 bound its own promoter and the promoters of the other three TFs in yeast-phase cells (Figure 6A). Tec1, Brg1 and Efg1 also bound the promoter regions of all four factors in hyphae (Figure 6A). Together with a recent genome-wide binding map of Efg1 in yeast phase [27], these data allow for the reconstruction of a partial map of the transcriptional circuit that underlies hyphal differentiation in *C. albicans* (Figure 6B).

## Discussion

In this study, we set out to dissect how the conserved Set3C histone deacetylase complex regulates hyphal differentiation in the human fungal pathogen *C. albicans*. Our data not only provide mechanistic and evolutionary insights into how chromatin deacetylation at the coding sequences regulates gene expression, but also allow for the characterization of the transcriptional circuitries underlying fungal morphogenesis. Remarkably, our data show that the activity of the transcriptional layer of gene regulation is fine-tuned by a second layer requiring chromatin modification.

### A transcription factor-chromatin modifier network controls *C. albicans* morphogenesis

To date, over a hundred genes have been implicated in *C. albicans* morphogenesis, including numerous conserved signaling cascades and transcription factors [6], [7], [8], [28]. However, a comprehensive understanding of how the underlying genetic circuit is organized and how it integrates outside stimuli is still lacking. Our data has led to surprising insights into the architecture of the hyphal regulatory circuit, whose simplified model is shown in Figure 6B. In this model, a core circuit is formed by four TFs, two of which are enriched in the yeast phase (*NRG1* and *EFG1*) and two are enriched in hyphae (*TEC1* and *BRG1*). The four factors form an interwoven network, whereby all four factors regulate themselves, as well as the other three factors. For practical reasons, we consider here the yeast-specific phase of the circuit as the ground state. In yeast cells, Nrg1 represses hyphal-specific genes, and cAMP/PKA signaling-dependent removal of Nrg1 is required for the induction of hyphal genes [10], [11], [12]. The relief of Nrg1 repression enables expression of hyphal-specific genes such as the TFs Brg1 and Tec1, which once expressed, repress the promoters of the yeast-specific regulators and reinforce their own expression (Figure 6A). This “excited” state of the circuit is subsequently responsible for the establishment of a normal hyphal transcription program. Indeed, all four TF genes are strong binding targets of the Set3C in their respective phases. In *set3*Δ/Δ cells, the circuit remains intact, but shows a “hyper-excited” state shortly after hyphal induction, that is reflected in the transcript levels of all four regulators (Figure 5, Figure 6B; Table S5). (It is, however, possible that the early surplus of *BRG1* is at least partially accountable for the transcriptional effect at the other three TF loci.) Further evidence for the “hyper-excited” state includes the elevated expression of verified Brg1 target transcripts such as *UME6* and *HGC1* (Figure S5). This model predicts that in *set3*Δ/Δ cells the circuit responds to weaker hypha-inducing stimuli than in wild type cells, which explains why *set3*Δ/Δ cells are hyperfilamentous under “intermediate” conditions (Figure S1, Figure 5C; [15]). The model receives further support by the finding that removal of one *BRG1* allele partially reverts the phenotype of *set3*Δ/Δ cells (Figure 5C). Thus, though many additional TFs are implicated in morphogenesis, we believe this simple circuit architecture is consistent with several key features of the differentiation process.

Recently, a similar, small transcription factor network composed of six core TFs was identified as the master network controlling biofilm formation of *C. albicans* [23]. Biofilms are formed on solid surfaces and indwelling medical devices by a population of diverse cell morphologies (including yeast, pseudohyphal and hyphal forms) that are embedded in an extracellular matrix. Remarkably, five out of the six biofilm regulators are Set3C targets (*TEC1*, *BRG1*, *EFG1*, *NDT80* and *ROB1*), and three factors are found in the hyphal regulatory circuit (*TEC1*, *BRG1* and *EFG1*). Hence, if the Set3C restricts the excitation state of the hyphal regulatory network, and there exists at least a partial overlap between the two regulatory circuits, the Set3C may also affect the output of the biofilm regulatory circuit and thus modulate biofilm formation. Strikingly, *set3*Δ/Δ cells produce a strong “rubbery” biofilm with visibly stronger mechanical properties when compared to wild type cells (Figure S6, Nobile and Johnson, unpublished data). These results strongly suggest that a kinetic control of transcriptional circuit components by the Set3C modulates biofilm formation, and likely constitutes a conserved regulatory mechanism underlying morphogenetic processes in *C. albicans*.

### Mechanistic and evolutionary aspects of the function of the Set3C

In *S. cerevisiae*, the Set3C binds to gene bodies where it deacetylates various residues of histones H3 and H4 [17]. The occupancy of the complex correlates with expression, and in *set3*Δ/Δ cells, galactose metabolic genes are turned on with a slower kinetics when compared to wild type after galactose induction, which suggests that the complex is required for gene induction [17]. In addition, Hos2, the catalytic subunit of the SetC3 binds at a few tRNA loci where it is necessary for efficient integration of Ty1 retrotransposons [29]. The *C. albicans* Set3C also decorates coding regions and tRNA loci, and its presence correlates with transcriptional activity, supporting the notion of conservation of Set3C localization being linked to active transcription. However, the genome-wide association analysis does not reveal how the recruitment of the complex affects transcription itself. In *S. cerevisiae*, the genetic removal of Set3 has only a marginal effect on genome-wide transcription [30]. Recently, a systematic study of chromatin-modifier mutants in *S. cerevisiae* revealed that several enzymes modulate the induction kinetics of their target genes rather than steady-state transcript levels [31]. Similarly, we detected transcript level differences at only 19% of the Set3C binding targets in *set3*Δ/Δ *C. albicans* cells. These genes mostly include the glycolytic genes that were all downregulated in the *set3*Δ/Δ mutant, arguing for a positive role of the Set3C in transcription (Figure 2), but the exact role of the Set3C is possibly determined by the context it is recruited. Thus, such a static view of the steady-state transcript levels is insufficient to predict precise function. Indeed, we detected 50% more *BRG1* mRNA in *set3*Δ/Δ cells than in wild type cells already after 10 minutes of hyphal induction, which implies that the Set3C can also exert repressive functions. This is in agreement with an early report where *set3*Δ/Δ diploid *S. cerevisiae* cells experienced premature activation of meiotic genes upon induction of sporulation [16], and indicates that both the context and timing of histone modifications contribute to the net transcriptional output [32]. Since in *set3*Δ/Δ cells, the TF-cluster genes were either “hyper-induced” or “hyper-repressed” during transient hyphal induction (Figure 5B), and several other Set3C targets showed altered transcription kinetics (Figure S5), we propose that the complex is also part of a conserved mechanism that creates a transient “transcriptional memory” at coding regions to buffer fast promoter changes. The mechanistic basis of opposing functions (repression and activation) could be that dynamic histone (de)acetylation affects nucleosome density at coding regions, while nucleosomes carry other modifications that directly affect transcription [33]. Indeed, we observed a slightly reduced nucleosome density at several target loci in *set3*Δ/Δ cells (data not shown).

Why would the chromatin at coding regions be used as such a relay platform? A detailed resolution of the transcriptomic response to several stresses in *S. cerevisiae* recently revealed that histone deacetylation at stress-responsive promoters by Rpd3 is required for normal induction and repression kinetics [34]. In our view, the utilization of coding region chromatin to adjust transcription kinetics is advantageous when several outside stimuli transmit potentially conflicting information to target promoters. In such cases, a transient decoupling of transcription from promoter input may provide sufficient time to shape the proper response to the environment or even prevent overshooting responses. In the cases of transcriptional circuits, the time needed to establish the new circuit output may be used to “decide” if the morphological conversion is in fact favorable under the given circumstances. Indeed, such a “test the waters” strategy was recently hypothesized to underlie white-opaque switching, another morphological switching process of *C. albicans* [35].

One of the most intriguing questions that arise based on these results is how the Set3C is selectively recruited to the coding sequences of its target genes. Localization is in fact so exclusive to gene bodies, that genomic segments encoding long 5′ untranslated regions (UTR) are completely devoid of ChIP-Seq signal (Figure S3C). In *S. cerevisiae*, recruitment of the Set3C has been linked to the recognition of H3K4me2 which decorates mainly coding regions [18], but most surprisingly, we found that genome-wide localization the *C. albicans* Set3C does not depend on dimethylation of H3K4 (Figure S7). Notably, despite the fact that Set3C occupancy correlates with expression of the corresponding locus, not all highly transcribed genes, for instance ribosomal protein genes, are Set3C targets (Figure 2). To rule out that this is caused by differences in RNA stability of the gene clusters, we examined RNAPII density [36], and found a similar correlation of Set3C enrichment with RNAPII density as with RNA level genome-wide (data not shown). This indicates that though Set3C recruitment is linked to active transcription by RNAPII, the recruitment signal stems from sequence-specific binding of transcription factors at the gene promoters. Our preliminary experiments indeed suggest that the promoter of a Set3C target gene is sufficient to direct the complex to exogenous gene bodies (data not shown). Although further experiments are necessary to dissect the mechanistic basis of the transmission of the recruitment information, we believe the transcriptional regulators either alone or more likely, in combination may direct posttranslational modification(s) of RNAPII, that contribute to Set3C recruitment once the polymerase reaches the coding region. Indeed, the plethora of modifications of the RNAPII C-terminal domain (CTD) has been postulated to constitute a “CTD code”, whereby specific CTD modifications orchestrate the binding of protein factors that affect RNA processing, RNAPII termination or histone modifications [37], [38], [39]. Thus, it is tantalizing to speculate that the Set3C and its homologues could serve not only as erasers of histone acetylation but also as readers of CTD modifications.

### Adaptation through phenotypic transitions in changing environments

During its coevolution with the human host, *C. albicans* had to adapt to various niches representing a wide spectrum of physical, chemical and immunological parameters. One of its adaptive strategies appears to be controlling phenotypic transitions through small, evolvable transcriptional circuits that are responsive to outside stimuli of broad amplitudes. Our model proposes that the switch between distinct circuit phases proceeds through an intermediate stage characterized by chromatin changes not just at promoters, but also at gene bodies. We postulate that such chromatin-overlayed transcriptional regulatory circuits underlie the morphological diversity of *C. albicans*, and most likely many other pleiomorphic fungal pathogens.

### Author note

We wish to point out that during the revision of this work, a report was published, demonstrating that the Set3C modulates transcription kinetics in response to carbon source shifts in *S. cerevisiae* [40]. This work shows that the yeast Set3C HDAC acts as an active repressor. Notably, indirect regulatory functions of Set3C can lead to positive regulation of target genes through a mechanism involving repression of overlapping non-coding RNAs [40].

## Materials and Methods

### Media and growth conditions

*C. albicans* strains were routinely cultured in YPD (2% Bacto Peptone, 1% yeast extract, 2% Dextrose). For solid media 2% agar was added. Yeast phase cultures were propagated at 30°C. To obtain exponentially growing yeast cells, single colonies were grown in YPD at 30°C overnight, diluted the next day to an optical density at 600 nm (OD_600_) of 0.1, and incubated on a rotary shaker for exactly five hours after which cultures reached OD_600_ = 0.8±0.05. For hyphal induction the cultures were split, one aliquot was washed once with distilled water and resuspended in prewarmed (37°C) YPD+20% fetal calf serum (FCS) of an equal volume of the starting culture. Induced cells were shaken at 37°C for 30 minutes, which provides enough time to induce several hypha-specific transcripts and it is before the first nuclear division occurring around 60 minutes after induction (data not shown). Cultures were optionally snap-frozen in liquid N_2_ after washing steps and stored at −80°C.

### Strain construction

The complete list of *C. albicans* strains, primers and plasmids used in this study are listed in Tables S1, 2 and S3, respectively. All strains were derived from SN152 [41]. The wild type (CAIF-100), *SET3*/*set3*Δ (DHCA401), *set3*Δ/Δ (DHCA402), *HOS2*/*hos2*Δ (DHCA405) and *hos2*Δ/Δ (DHCA406) strains were previously described [15], [42]. *SET1*, *BRG1* and *TEC1* alleles were deleted using the fusion PCR strategy with the *C.d.ARG4* and *NAT1* markers [15], [41]. *NRG1* and *SET3* alleles were deleted using the SAT1 flipping strategy [15], [43]. Epitope-tagging constructs were created using the fusion PCR strategy [44] with the “tag-marker” donor plasmids described in Table S3. The Efg1-myc (CJN1781), Brg1-myc (CJN1734) and *tec1*Δ/Δ (CJN2320) strains were previously described [23]. The construct for targeted replacement of *NRG1* with *GFP* was also created by fusion PCR. Transformation was performed via electroporation as described [43]. Correct genomic integration was verified with PCR and immunoblotting (for epitope tags).

### Immunoprecipitation (IP)

IP was performed essentially as described [18] with modifications. Exponentially growing cells were harvested by centrifugation and washed twice with distilled water. For Set3-HA and Hos2-3HA IPs (Figure 1B and Figure S1B) cells were resuspended in 300 µl stringent lysis buffer (phosphate-buffered saline (PBS) with 300 mM NaCl, 1% Triton X-100, 2 mM DTT and protease inhibitors). After addition of around 200 µl glass beads (425–600 µm, Sigma), cells were lysed at 5 m/s for 45 seconds 5 times on a FastPrep instrument (MP Biomedicals). Samples were chilled on ice for five minutes between cycles. Tubes were then centrifuged at 14000 g for 5 min at 4°C, the supernatant (around 300 µl) was transferred to a fresh tube and diluted to 1 ml with stringent lysis buffer. Whole cell extracts were incubated at 4°C overnight with agarose beads covalently bound to the 3F10 clone anti-HA antibody (Roche) with the amount of beads previously titrated to the amount of antigen in the respective amounts of extracts. The next day beads were washed six times with stringent lysis buffer and bound complexes were resolved by SDS-PAGE. For the Set3-3HA-histone co-IPs (Figure S7) the same protocol was followed, except that a mild lysis buffer (10 mM Tris-HCl pH 8.0, 150 mM NaCl, 0.1% Nonidet P-40 and protease inhibitors) was used throughout the whole procedure and beads were washed only four times after immunoprecipitation. The antibodies used in the Western blot detection included anti-HA (3F10, Roche), anti-myc (ab32, Abcam) anti-H3 (ab1791, Abcam), anti-H4 (ab10158, Abcam) and anti-tubulin (DM1A, Sigma).

### RNA isolation and cDNA synthesis

RNA was isolated by the hot phenol method [44] after which RNA was further purified by the SV Total RNA isolation system (Promega) according to the manufacturer's instructions. For qPCR, 500 ng total RNA was reverse-transcribed using the AMV reverse transcription system (Promega), and the diluted first strand cDNA was directly used as template. For RNA-Seq, 5 µg total RNA was depleted of rRNAs using the RiboMinus kit (Invitrogen) and cDNA was synthesized and amplified with the SMARTer cDNA synthesis kit (Clonetech) according to the manufacturer's instructions. 5 µg cDNA was purified with phenol∶chloroform∶isoamylalcohol extraction (PCI), precipitated in 70% ethanol at −20°C overnight, washed once with 70% ethanol, dissolved in distilled water, and was used as a template for library preparation. RNA quality was controlled with the BioAnalyzer (Agilent) during the whole procedure.

### Chromatin immunoprecipitation (ChIP)

ChIP was performed essentially as described [44] with several modifications. Cultures were crosslinked by the addition of formaldehyde at a final concentration of 1% for 15 minutes at room temperature. Crosslinking was quenched by the addition of 125 mM glycine for 5 minutes. Cells were washed twice with ice-cold Tris-buffered saline (TBS) and pellets were frozen in liquid N_2_. Pellets corresponding to 80 OD_600_ cells were resuspended in 480 µl ice-cold ChIP lysis buffer (50 mM HEPES/KOH pH 7.5, 140 mM NaCl, 1 mM EDTA, 1% Triton X-100, 0.1% Na-deoxycholate, protease inhibitors). After addition of around 500 µl glass beads (425–600 µm, Sigma), cells were broken at 6 m/s for 60 seconds 8 times on a FastPrep instrument (MP Biomedicals). Samples were cooled on ice for five minutes between cycles. The bottom of the tubes was punctured with a 27^3^/_4_G needle and lysates were collected by centrifugation at 1500 g for 1 min at 4°C into a fresh tube. The lysates were diluted to 2.4 ml with ChIP lysis buffer. Aliquots of 300 µl were sonicated 15 times with 30s ON/30s OFF cycles at high setting on a Bioruptor (Diagenode). After sonication the aliquots were combined and centrifuged once at 14000 g for 5 min at 4°C. Supernatants were collected and used as input chromatin lysates. Set3-9myc and Hos2-9myc ChIPs were performed using EZ-View anti-myc coupled agarose beads (Sigma). After overnight incubation at 4°C washing and DNA purification was performed exactly as described [44]. For histone ChIPs (on Figure 1E, Figure S4C, Figure S7F) anti-acetyl H4 (06-598, Millipore), anti-H3 (ab1791, Abcam) and anti-H3K4me2 (07-030, Millipore) were used. Brg1-myc and Efg1-myc ChIP was performed with a commercial anti-myc antibody exactly as described [23]. Nrg1-3HA ChIP was performed with an anti-HA antibody (ab9110, Abcam). Tec1 ChIP was performed with a custom anti-Tec1 antibody [23]. After overnight incubation at 4°C, ProteinG-coupled Dynabeads (Invitrogen) were added for 2 h at 4°C, and subsequent washing and DNA purification steps were performed exactly as described [44]. The sonication settings typically resulted in fragments sizes mostly around 200–300 bp, which was controlled by agarose gelelectrophoresis of purified input samples. All ChIP experiments were carried out at least with three biological replicate cultures.

### RNA–Sequencing (RNA–Seq) and analysis

Sequencing of fragmented cDNA was carried out on a HiSeq 2000 instrument (Illumina) at GATC Biotech AG (Konstanz, Germany). Three biological replicates of wild type and *set3*Δ/Δ cells in both yeast and hyphal phases were included. The resulting 51 base reads were mapped onto the Assembly 21 of the *C. albicans* genome containing only the coding sequences using TopHat with default parameters, and allowing only for uniquely mapping reads [45]. Fragment Per Kilobase in a Million mapped reads (FPKM) values were calculated with Cufflinks, including quartile normalization (removing top 25% of genes from the FPKM denominator) and bias correction [46]. The transcript coordinates were fixed as in the annotation of the coding sequence assembly. For transcriptome analyses on Figure 2B and Figure 3B and the right panel of Figure 2A, only genes with a mean coverage of at least five nucleotides per base were included. Mean coverage was calculated as an average of the three biological replicates for each genotype or phase. For the transcriptome analysis on Figure 3E the same mean coverage cutoff (>5 nt/base) was used, but only for the wild type yeast and hyphal RNA-Seq samples. The complete RNA-Seq dataset is found in Table S6.

### ChIP–Sequencing (ChIP–Seq) and analysis

ChIP libraries were sequenced on a GAIIx platform (Illumina). Three biological replicates of the Set3-9myc ChIP in wild type and *set1*Δ/Δ backgrounds in yeast phase cells were sequenced, with two biological replicates of Set3-9myc ChIP and Hos2-9myc ChIP in hyphal phase cells and two biological replicates of Hos2-9myc ChIP in yeast phase cells. ChIP material of six biological replicates of the untagged control strain in the yeast phase was pooled prior to library preparation. One input sample of each genotype in each morphological phase was also sequenced. Samples were multiplexed with custom adapters. Reads (31–32 base) were mapped onto the chromosomal Assembly 21 of the *C. albicans* genome using Bowtie, allowing only for uniquely mapping reads [47] (Figure S2). Peak calling was performed by MACS with mfold = 2 and effective genome size = 14.324.316 bp parameters [21]. With a peak calling method such as MACS, detected peak coordinates rely on arbitrary thresholds and can vary even between replicate samples, and peak positions cannot be adjusted. Therefore, we developed a Read Per Kilobase in a Million mapped reads (RPKM) pipeline for quantifying ChIP-Seq enrichment within gene bodies. RPKM measures the enrichment of sequence reads within fixed chromosomal coordinates and is generally used to quantify RNA species in RNA-Seq experiments when the precise transcript coordinates are known [48]. The RPKM values of all open reading frames (ORFs) were calculated using their default chromosomal coordinates in ORF Assembly 21. Overlapping genes were removed from further analyses. Since tRNA genes are relatively short (80–120 bp), 20 bases up- and downstream of their chromosomal coordinates were added to define tRNA-cluster positions, which were used in the RPKM calculation. If two adjacent tRNA genes were closer than 20 bases, they were included in the same cluster. Overall, RPKM(ChIP)/RPKM(input) values correlated with the MACS fold enrichment parameter in the same sample (Figure S3B). Hence, RPKM is a suitable quantitative readout of our peak signals. Enriched genes were initially defined as having an RPKM(ChIP)/RPKM(input) ≥2 with both RPKM values ≥10 (“targets” on Figure 1C). Since a few ORFs showed minor enrichment in the untagged control ChIP-Seq sample (Figure 1C and Figure S2), the RPKM ChIP values were subsequently normalized against the RPKM ChIP values of the untagged control instead of the respective input samples. Further normalization against the input samples for the comparison of genotypes was not necessary, because RPKM values of all input samples showed a high pairwise correlation (*r*>0.9, Pearson's correlation, not shown). We defined the Set3C target set as an ORF having at least 2-fold Set3 or Hos2 enrichment and at least 1.5-fold enrichment of the other (blue box, Figure 1D), or a tRNA having at least 1.5-fold enrichment of both (Figure 1D). For the genome-wide enrichment analyses on Figure 1D, Figure 3C, and Figure S7 only genes with an average RPKM≥5 in both genotypes or phases were included. On Figure 3C only two biological replicates (R2 and R3) of the Set3-9myc yeast phase ChIP-Seq samples were included. Mapped reads were extended with the length of the MACS *d* parameter (∼150 bp) prior to visualization. The complete ChIP-Seq dataset including chromosomal coordinates and RPKM values of all ORFs and tRNA clusters is found in Table S4.

### Data availability

Data have been deposited at the Gene Expression Omnibus (GEO) under accession number GSE38427.

## Supporting Information

Figure S1 The *C. albicans* Set3C is a conserved nuclear complex. (A) Phenotypic analysis of *C. albicans* strains carrying epitope-tagged *SET3* and *HOS2* alleles. Colonies were photographed after incubation on YPD medium at 37°C for three days. Scale bar corresponds to 2 mm. (B) Identification of Set3 and Hos2 interaction partners with immunoprecipitation followed by mass spectrometry. The molecular weights of subunits based on homology to *S. cerevisiae* are shown on the left panel. Set3-3HA and Hos2-3HA were immunoprecipitated from whole cell extracts and the bound complexes were resolved on SDS-PAGE. The bands indicated by an arrow on the Silver stained gel were isolated and identified by mass spectrometry. Aa: aminoacid Sc: *S. cerevisiae*, Ca: *C. albicans*, MW: molecular weight (kDa). (C) GFP-tagged Hos2 shows nuclear localization. Nuclei were stained with Hoechst solution. Scale bar corresponds to 10 µm.
(PDF)

Figure S2 Snapshot of the Genome Browser. Reads of the indicated ChIP-Seq samples were mapped on the Assembly 21 of the *C. albicans* genome. Reads were extended with the length of the MACS *d* parameter (∼150 bp) prior to visualization. The right arm of chromosome 2 is shown. MRS indicates the Major Repeat Sequence, a repetitive element.
(PDF)

Figure S3 Set3 and Hos2 primarily localize to coding regions and tRNA loci. (A) MACS analysis of one representative Set3 and Hos2 ChIP-Seq experiment. Peaks were identified based on enrichment over the respective input samples as described at the Materials and Methods. Only the peaks with P<10^−5^ were included. On the left panel, the total number of bases in the reads that map to the peak regions is shown (first column), as well as the percentage of the bases located within coding regions, promoters (1000 bases upstream of the start codon), tRNA loci or intergenic regions (all positions not included in the other three). The composition of the genome as percentage of the four categories is also displayed (third column), as well as the enrichment of bases in each category in the peaks over the genomic composition (fourth column). On the right panel, the percentage of peaks having a summit position located within the four genomic features is shown. The Assembly 21 of the *C. albicans* genome was used to determine the genome composition. (B) Correlation of MACS fold enrichment values with the enrichment measured by the RPKM method in one representative Set3 and Hos2 ChIP-Seq experiment. Only the peaks that overlap with gene bodies and with P<10^−30^ were included. “*r*” denotes a Pearson's correlation coefficient. (C) Genomic segments encoding long 5′ untranslated regions (UTR) are devoid of Set3C ChIP-Seq signal. A snapshot of the Genome Browser is shown. The coding region is displayed in a blue box. RNAPII ChIP-chip track was taken from [36].
(PDF)

Figure S4 Set3C is associated with active transcription. (A) Set3C binding correlates with transcriptional activity genome-wide. Average log2 FPKM values of three RNA-Seq experiments are plotted against Hos2 enrichment. Each dot represents the moving average of 100 genes, and sliding step size is one gene, exactly as described at Figure 2 in [17]. “*r*” denotes a Pearson's correlation coefficient. (B) qRT-PCR validation of the downregulation of hexose catabolic (glycolytic) genes in *set3*Δ/Δ cells. Data are shown as mean+SD of three independent experiments. Statistical significance was determined by two-tailed t-test relative to the control values. *P<0.05, **P<0.01, ***P<0.001. (C) Histone H4 acetalytion is increased at many target loci in *set3*Δ/Δ cells. ChIP experiments were performed with antibodies against acetylated histone H4 and the C-terminus of histone H3. The qPCR probes were designed to amplify a fragment at the 5′end of the respective genes. The qPCR values at the probe positions were normalized to a fragment of the telomere of Chromosome 7. The ratio of the signal of the acetylated H4 ChIP and H3 ChIP is shown on the y-axis. Data are shown as mean+SD of two independent experiments. Statistical significance was determined by two-tailed t-test relative to the control values. *P<0.05, **P<0.01, ***P<0.001. (D) Acetylation of H4 at the coding region correlates with RNA expression level. Hexose catabolism genes are colored red, TF genes are colored black. “*r*” denotes a Pearson's correlation coefficient.
(PDF)

Figure S5 qRT-PCR analysis of additional transcripts in differentiating wild-type and *set3*Δ/Δ cells. Transcripts in wild-type and *set3*Δ/Δ yeast cells induced to differentiate into hyphae 10, 20, 30 and 60 minutes after induction are shown exactly as described on Figure 5B. The genes are grouped whether they are Set3C binding targets in either morphological phase. *UME6* and *HGC1* (in blue box) are major binding targets of the Brg1 transcription factor in hyphae [12], and their elevated expression at 30 and 60 min post induction in *set3*Δ/Δ cells suggests Brg1 hyperactivity. Data are shown as mean+SD of four independent experiments. Statistical significance was determined by two-tailed t-test. *P<0.05, **P<0.01, ***P<0.001.
(PDF)

Figure S6 Phenotypic comparison of wild-type and *set3*Δ/Δ biofilms. Biofilm growth assays were carried out in Spider medium by growing the biofilm directly on the bottom of 6-well polystyrene plates, as follows. Strains were grown overnight in YPD at 30°C, and diluted to an OD_600_ of 0.5 in 4 ml Spider medium for each well of the 6-well plate. The 6-well plate was then incubated at 37°C for 90 min at 200 rpm agitation for initial adhesion of cells in an ELMI digital thermostatic shaker. The plates were washed with 4 ml PBS, and 4 ml of fresh Spider medium was added. The plate was incubated at 37°C for an additional 48 h at 200 rpm agitation to allow biofilm formation. *set3*Δ/Δ cells form a strong “rubbery” biofilm that can be completely removed by the tip of a Pasteur pipette.
(PDF)

Figure S7 H3K4 methylation is dispensable for Set3C recruitment. (A) Alignment of the sequence of the CaSet3 PHD domain to other PHD domains. The eight residues forming the Zn-finger are framed in blue boxes. The residues implicated in the binding of methylated H3K4 are highlighted with arrowheads. (B) Comparative analysis of the predicted structure of the CaSet3 PHD domain. The left side shows the crystal structure of the PHD domain of HsBPTF adapted from [49]. The PHD domain is colored blue, and the eight N-terminal residues of histone H3 are colored magenta. The residues forming the binding pocket are colored red, and the aspartic acid coordinating H3R2 is yellow. The residues H3R2 and H3K4 are colored green. The right side shows the structural model of the CaSet3 PHD overlayed on the crystal structure on the left using Modeller [50] with identical color-coding. (C) Set3 co-purifies with nucleosomal histones *in vitro*. Set3-3HA was immunprecipitated from whole cell extracts and the interaction is probed by Western blot detection of histones H3 and H4. The interaction is lost in *set1*Δ/Δ cells that lack H3K4 methylation. (D) Set3 occupancy is not lost in *set1*Δ/Δ cells *in vivo*. Set3 enrichment values were measured by the RPKM method of three independent ChIP-Seq experiments. Each dot corresponds to one gene. Set3C targets not bound in *set1*Δ/Δ cells are highlighted blue, de novo Set3C targets in *set1*Δ/Δ cells are highlighted red. “*r*” denotes a Pearson's correlation coefficient. (E) Validation of a de novo Set3C binding target orf19.7502 in *set1*Δ/Δ cells with qPCR. On the top panel a snapshot of the read density profiles from the Genome Browser is shown with the chromosomal coordinates. Data are shown as mean+SD of three independent experiments. Statistical significance was determined by two-tailed t-test. *P<0.05, **P<0.01, ***P<0.001. (F) Deacetylation of histone H4 by Set3C is independent of H3K4 methylation at the *PFK1* locus. Top panel: ChIP experiments were performed with antibodies against acetylated histone H4 and the C-terminus of histone H3. The qPCR values at the probe positions were normalized to a fragment of the telomere of Chromosome 7. Bottom panel: Histone H3K4 dimethylation is unaffected in *set3*Δ/Δ cells. ChIP experiments were performed with antibodies against H3K4me2 and the C-terminus of histone H3. The qPCR values at the probe positions were normalized to a fragment of *ADE2*. We failed to retrieve usable ChIP DNA from *set1*Δ/Δ with the H3K4me2 antibody. Data are shown as mean+SD of two independent experiments. Statistical significance was determined by two-tailed t-test relative to the control values. *P<0.05, **P<0.01, ***P<0.001. n.d.: not determined.
(PDF)

Table S1 List of *C. albicans* strains used in the study.
(DOC)

Table S2 List of primers used in the study.
(DOC)

Table S3 List of plasmids used in the study.
(DOC)

Table S4 ChIP-Seq RPKM values in all samples for all Assembly 21 ORFs and tRNA clusters.
(XLS)

Table S5 Set3C target gene set in yeast and hyphal phases.
(XLS)

Table S6 FPKM and coverage values for all RNA-Seq samples.
(XLS)
