# Supplementary figures and images for: A Histone Deacetylase Adjusts Transcription Kinetics at Coding Sequences during Candida albicans Morphogenesis
Source: PLoS Genet. 2012 Dec 6;8(12):e1003118. doi: 10.1371/journal.pgen.1003118 (PMC3516536; doi:10.1371/journal.pgen.1003118)

Figure S1

A

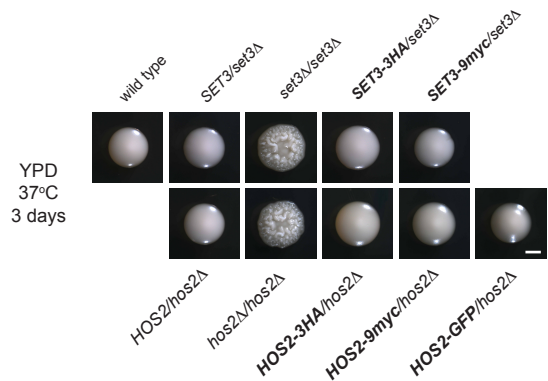

B

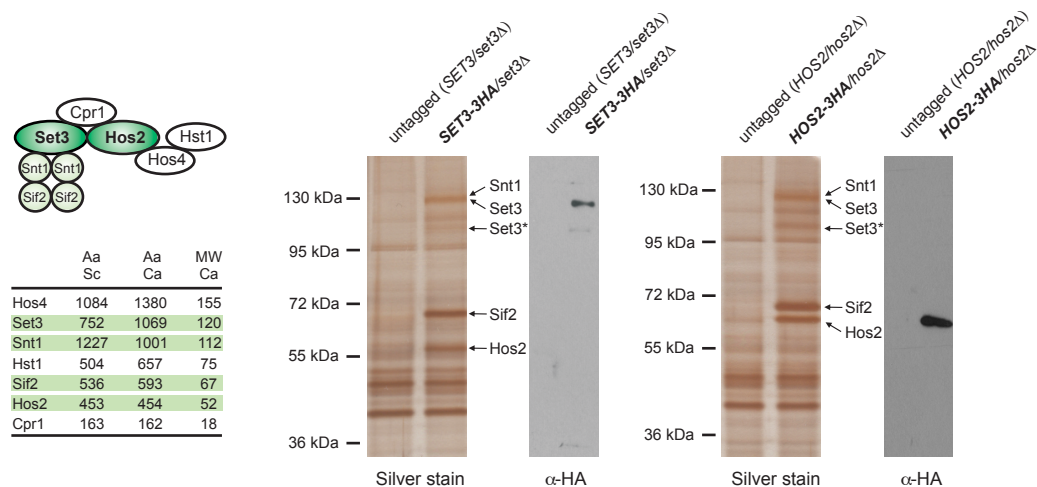

C

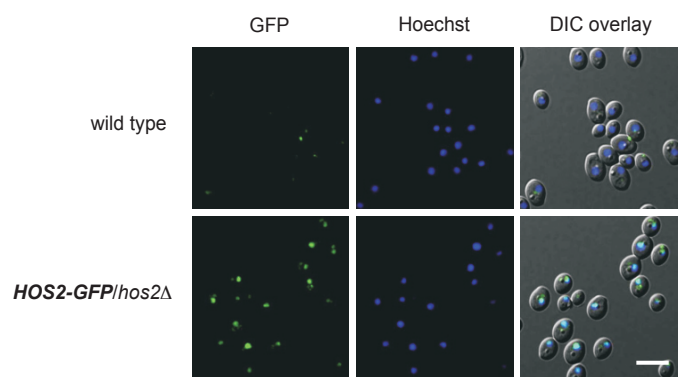

Supplement: Figure S1 — The C. albicans Set3C is a conserved nuclear complex. (A) Phenotypic analysis of C. albicans strains carrying epitope-tagged SET3 and HOS2 alleles. Colonies were photographed after incubation on YPD medium at 37°C for three days. Scale bar corresponds to 2 mm. (B) Identification of Set3 and Hos2 interaction partners with immunoprecipitation followed by mass spectrometry. The molecular weights of subunits based on homology to S. cerevisiae are shown on the left panel. Set3-3HA and Hos2-3HA were immunoprecipitated from whole cell extracts and the bound complexes were resolved on SDS-PAGE. The bands indicated by an arrow on the Silver stained gel were isolated and identified by mass spectrometry. Aa: aminoacid Sc: S. cerevisiae, Ca: C. albicans, MW: molecular weight (kDa). (C) GFP-tagged Hos2 shows nuclear localization. Nuclei were stained with Hoechst solution. Scale bar corresponds to 10 µm. (PDF) [file pgen.1003118.s001.pdf]

Figure S2

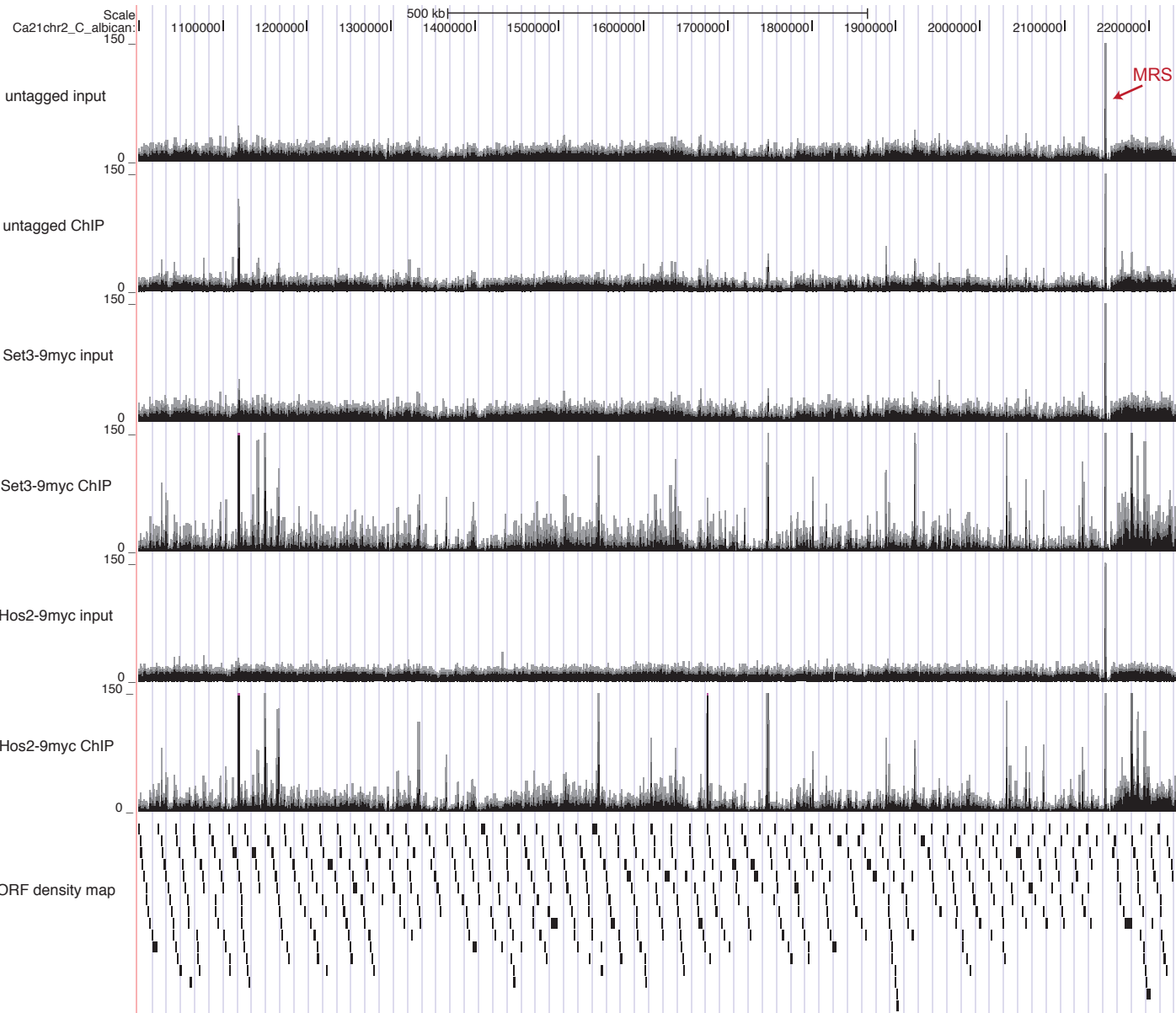

Supplement: Figure S2 — Snapshot of the Genome Browser. Reads of the indicated ChIP-Seq samples were mapped on the Assembly 21 of the C. albicans genome. Reads were extended with the length of the MACS d parameter (∼150 bp) prior to visualization. The right arm of chromosome 2 is shown. MRS indicates the Major Repeat Sequence, a repetitive element. (PDF) [file pgen.1003118.s002.pdf]

Figure S4

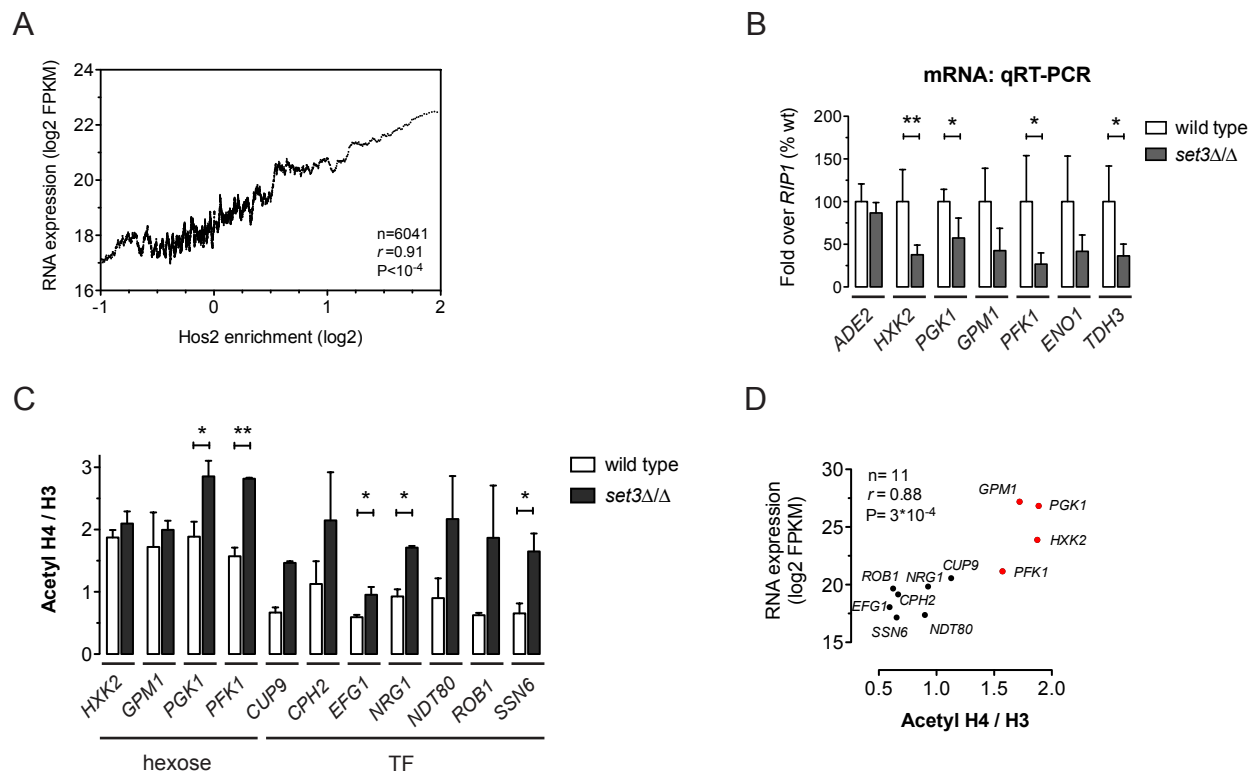

Supplement: Figure S4 — Set3C is associated with active transcription. (A) Set3C binding correlates with transcriptional activity genome-wide. Average log2 FPKM values of three RNA-Seq experiments are plotted against Hos2 enrichment. Each dot represents the moving average of 100 genes, and sliding step size is one gene, exactly as described at Figure 2 in [17]. “r” denotes a Pearson's correlation coefficient. (B) qRT-PCR validation of the downregulation of hexose catabolic (glycolytic) genes in set3Δ/Δ cells. Data are shown as mean+SD of three independent experiments. Statistical significance was determined by two-tailed t-test relative to the control values. *P<0.05, **P<0.01, ***P<0.001. (C) Histone H4 acetalytion is increased at many target loci in set3Δ/Δ cells. ChIP experiments were performed with antibodies against acetylated histone H4 and the C-terminus of histone H3. The qPCR probes were designed to amplify a fragment at the 5′end of the respective genes. The qPCR values at the probe positions were normalized to a fragment of the telomere of Chromosome 7. The ratio of the signal of the acetylated H4 ChIP and H3 ChIP is shown on the y-axis. Data are shown as mean+SD of two independent experiments. Statistical significance was determined by two-tailed t-test relative to the control values. *P<0.05, **P<0.01, ***P<0.001. (D) Acetylation of H4 at the coding region correlates with RNA expression level. Hexose catabolism genes are colored red, TF genes are colored black. “r” denotes a Pearson's correlation coefficient. (PDF) [file pgen.1003118.s004.pdf]

Figure S5

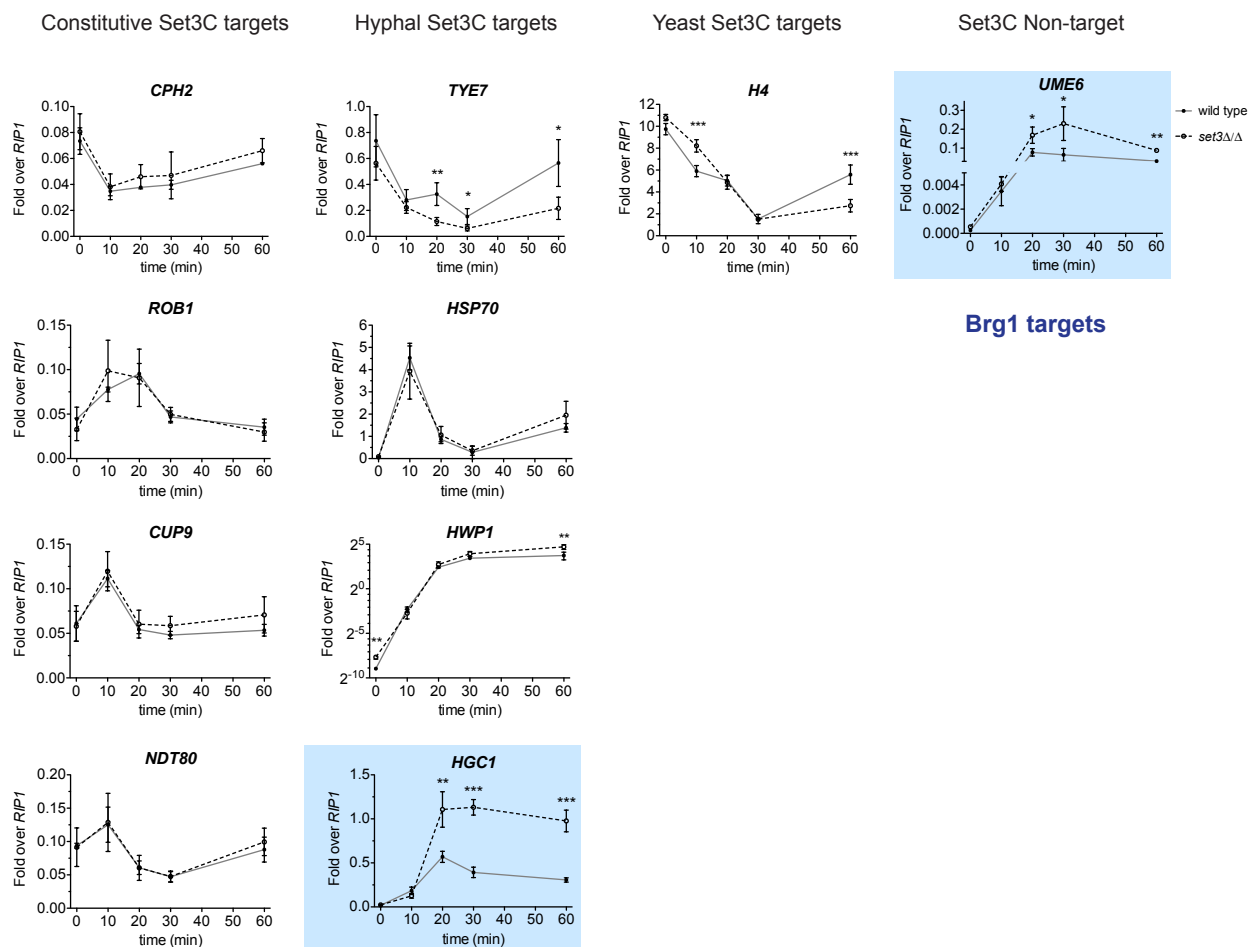

Supplement: Figure S5 — qRT-PCR analysis of additional transcripts in differentiating wild-type and set3Δ/Δ cells. Transcripts in wild-type and set3Δ/Δ yeast cells induced to differentiate into hyphae 10, 20, 30 and 60 minutes after induction are shown exactly as described on Figure 5B. The genes are grouped whether they are Set3C binding targets in either morphological phase. UME6 and HGC1 (in blue box) are major binding targets of the Brg1 transcription factor in hyphae [12], and their elevated expression at 30 and 60 min post induction in set3Δ/Δ cells suggests Brg1 hyperactivity. Data are shown as mean+SD of four independent experiments. Statistical significance was determined by two-tailed t-test. *P<0.05, **P<0.01, ***P<0.001. (PDF) [file pgen.1003118.s005.pdf]

Figure S6

wild type biofilm

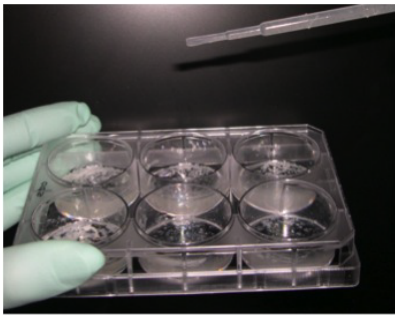

*set3Δ/set3Δ* biofilm

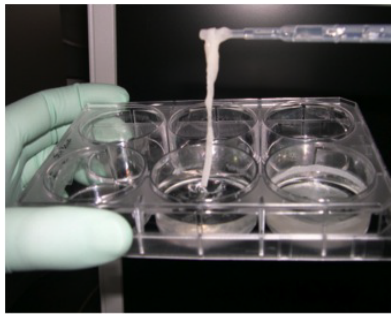

Supplement: Figure S6 — Phenotypic comparison of wild-type and set3Δ/Δ biofilms. Biofilm growth assays were carried out in Spider medium by growing the biofilm directly on the bottom of 6-well polystyrene plates, as follows. Strains were grown overnight in YPD at 30°C, and diluted to an OD600 of 0.5 in 4 ml Spider medium for each well of the 6-well plate. The 6-well plate was then incubated at 37°C for 90 min at 200 rpm agitation for initial adhesion of cells in an ELMI digital thermostatic shaker. The plates were washed with 4 ml PBS, and 4 ml of fresh Spider medium was added. The plate was incubated at 37°C for an additional 48 h at 200 rpm agitation to allow biofilm formation. set3Δ/Δ cells form a strong “rubbery” biofilm that can be completely removed by the tip of a Pasteur pipette. (PDF) [file pgen.1003118.s006.pdf]

Figure S7

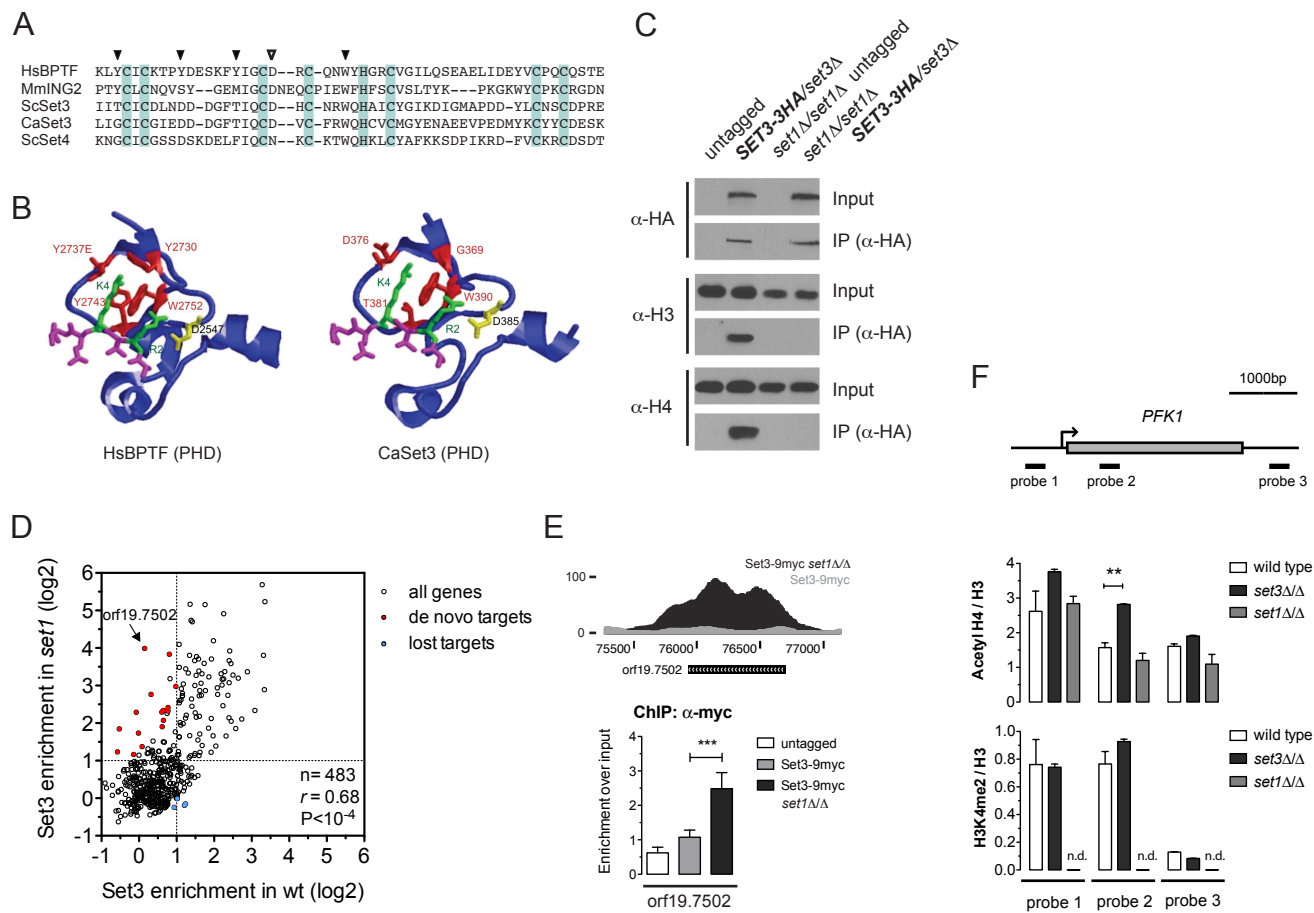

Supplement: Figure S7 — H3K4 methylation is dispensable for Set3C recruitment. (A) Alignment of the sequence of the CaSet3 PHD domain to other PHD domains. The eight residues forming the Zn-finger are framed in blue boxes. The residues implicated in the binding of methylated H3K4 are highlighted with arrowheads. (B) Comparative analysis of the predicted structure of the CaSet3 PHD domain. The left side shows the crystal structure of the PHD domain of HsBPTF adapted from [49]. The PHD domain is colored blue, and the eight N-terminal residues of histone H3 are colored magenta. The residues forming the binding pocket are colored red, and the aspartic acid coordinating H3R2 is yellow. The residues H3R2 and H3K4 are colored green. The right side shows the structural model of the CaSet3 PHD overlayed on the crystal structure on the left using Modeller [50] with identical color-coding. (C) Set3 co-purifies with nucleosomal histones in vitro. Set3-3HA was immunprecipitated from whole cell extracts and the interaction is probed by Western blot detection of histones H3 and H4. The interaction is lost in set1Δ/Δ cells that lack H3K4 methylation. (D) Set3 occupancy is not lost in set1Δ/Δ cells in vivo. Set3 enrichment values were measured by the RPKM method of three independent ChIP-Seq experiments. Each dot corresponds to one gene. Set3C targets not bound in set1Δ/Δ cells are highlighted blue, de novo Set3C targets in set1Δ/Δ cells are highlighted red. “r” denotes a Pearson's correlation coefficient. (E) Validation of a de novo Set3C binding target orf19.7502 in set1Δ/Δ cells with qPCR. On the top panel a snapshot of the read density profiles from the Genome Browser is shown with the chromosomal coordinates. Data are shown as mean+SD of three independent experiments. Statistical significance was determined by two-tailed t-test. *P<0.05, **P<0.01, ***P<0.001. (F) Deacetylation of histone H4 by Set3C is independent of H3K4 methylation at the PFK1 locus. Top panel: ChIP experiments were performed with a [file pgen.1003118.s007.pdf]
