# Supplementary material for: A Histone Deacetylase Adjusts Transcription Kinetics at Coding Sequences during Candida albicans Morphogenesis
Source: PLoS Genet. 2012 Dec 6;8(12):e1003118. doi: 10.1371/journal.pgen.1003118 (PMC3516536; doi:10.1371/journal.pgen.1003118)

Figure S3

A

ChIP: Set3-9myc

MACS PEAKS	total bases	%	% of region in genome	enrichment (fold)
Coding sequence	1815095	86.69	62.96	1.38
Promoter (-1000bp)	177023	8.45	22.77	0.37
tRNA	5437	0.26	0.09	2.81
Intergenic	96273	4.60	14.18	0.32

PEAK SUMMITS	# of summits	% of total
Coding sequence	1455	92.92
Promoter (-1000bp)	32	2.04
tRNA	39	2.49
Intergenic	40	2.55

ChIP: Hos2-9myc

MACS PEAKS	total bases	%	% of region in genome	enrichment (fold)
Coding sequence	1983187	84.57	62.96	1.34
Promoter (-1000bp)	252850	10.78	22.77	0.47
tRNA	5317	0.23	0.09	2.46
Intergenic	103578	4.42	14.18	0.31

PEAK SUMMITS	# of summits	% of total
Coding sequence	1270	94.78
Promoter (-1000bp)	29	2.16
tRNA	21	1.57
Intergenic	20	1.49

B

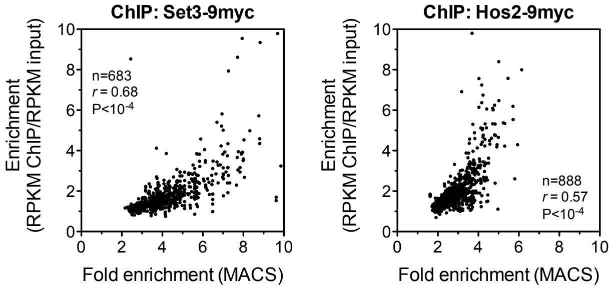

C

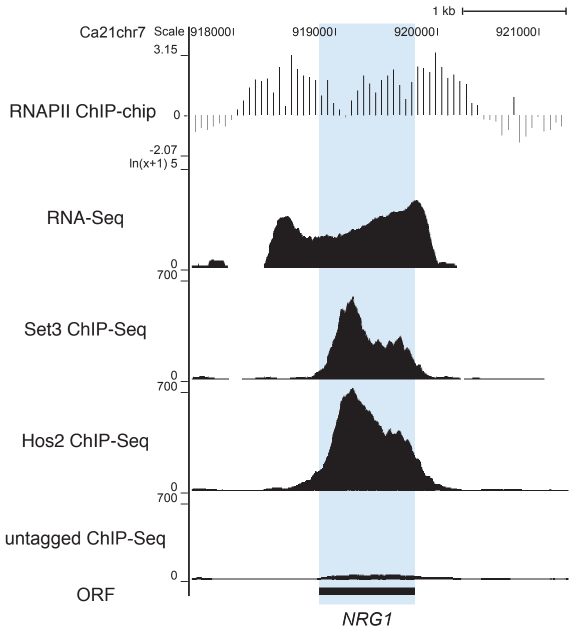

Supplement: Figure S3 — Set3 and Hos2 primarily localize to coding regions and tRNA loci. (A) MACS analysis of one representative Set3 and Hos2 ChIP-Seq experiment. Peaks were identified based on enrichment over the respective input samples as described at the Materials and Methods. Only the peaks with P<10−5 were included. On the left panel, the total number of bases in the reads that map to the peak regions is shown (first column), as well as the percentage of the bases located within coding regions, promoters (1000 bases upstream of the start codon), tRNA loci or intergenic regions (all positions not included in the other three). The composition of the genome as percentage of the four categories is also displayed (third column), as well as the enrichment of bases in each category in the peaks over the genomic composition (fourth column). On the right panel, the percentage of peaks having a summit position located within the four genomic features is shown. The Assembly 21 of the C. albicans genome was used to determine the genome composition. (B) Correlation of MACS fold enrichment values with the enrichment measured by the RPKM method in one representative Set3 and Hos2 ChIP-Seq experiment. Only the peaks that overlap with gene bodies and with P<10−30 were included. “r” denotes a Pearson's correlation coefficient. (C) Genomic segments encoding long 5′ untranslated regions (UTR) are devoid of Set3C ChIP-Seq signal. A snapshot of the Genome Browser is shown. The coding region is displayed in a blue box. RNAPII ChIP-chip track was taken from [36]. (PDF) [file pgen.1003118.s003.pdf]
