# Supplementary material for: A Histone Deacetylase Adjusts Transcription Kinetics at Coding Sequences during Candida albicans Morphogenesis
Source: PLoS Genet. 2012 Dec 6;8(12):e1003118. doi: 10.1371/journal.pgen.1003118 (PMC3516536; doi:10.1371/journal.pgen.1003118)
Supplement: Table S1 — List of C. albicans strains used in the study. (DOC) [file pgen.1003118.s008.doc]

**Table S1.** ***C. albicans*** **strains used in this study**

| **Description** | **Name** | ***MTL*** | **Parent** | **Genotype** | **Reference** |
| --- | --- | --- | --- | --- | --- |
| Wild type | SC5314 | **a**/α | Clinical isolate |  | [1] |
| Wild type | SN152 | **a**/α | SC5314 | *arg4*Δ/*arg4*Δ *his1*Δ/*his1*Δ *leu2*Δ/*leu2*Δ *URA3*/*ura3*Δ::*λimm^434^* *IRO1*/*iro1*Δ::*λimm^434^* | [2] |
| Wild type  (“untagged”) | CAIF-100 | **a**/α | SN152 | *arg4*Δ/*arg4*Δ *his1*Δ/*his1*Δ::*C.d.HIS1 leu2*Δ/*leu2*Δ::*C.m.LEU2 URA3*/*ura3*Δ::*λimm^434^* *IRO1*/*iro1*Δ::*λimm^434^* | [3] |
| *SET3*/*set3*Δ | DHCA401 | **a**/α | SN152 | *SET3*/*set3*Δ::*C.d.HIS1* | [4] |
| *set3*Δ/*set3*Δ | DHCA402 | **a**/α | SN152 | *set3*Δ::*C.d.HIS1*/*set3*Δ::*C.m.LEU2* | [4] |
| *SET3-3HA*/*set3*Δ | DHCA501 | **a**/α | DHCA401 | *SET3-3HA-NAT1*/*set3*Δ::*C.d.HIS1* | This study |
| *SET3-9myc*/*set3*Δ | DHCA502 | **a**/α | DHCA401 | *SET3-9myc-C.m.LEU2*/*set3*Δ::*C.d.HIS1* | This study |
| *HOS2*/*hos2*Δ | DHCA405 | **a**/α | SN152 | *HOS2*/*hos2*Δ::*C.d.HIS1* | [4] |
| *hos2*Δ/*hos2*Δ | DHCA406 | **a**/α | SN152 | *hos2*Δ::*C.d.HIS1*/*hos2*Δ::*C.m.LEU2* | [4] |
| *HOS2-3HA*/*hos2*Δ | DHCA503 | **a**/α | DHCA405 | *HOS2-3HA-NAT1*/*hos2*Δ::*C.d.HIS1* | This study |
| *HOS2-9myc*/*hos2*Δ | DHCA504 | **a**/α | DHCA405 | *HOS2-9myc-C.m.LEU2*/*hos2*Δ::*C.d.HIS1* | This study |
| *HOS2-GFP*/*hos2*Δ | DHCA505 | **a**/α | DHCA405 | *HOS2-GFP-NAT1*/*hos2*Δ::*C.d.HIS1* | This study |
| *set1*Δ/*set1*Δ | DHCA226 | **a**/**a** | DHCA202 | *set1*Δ::*C.d.HIS1*/*set1*Δ::*C.m.LEU2* | [5] |
| *set1*Δ/*set1*Δ *SET3*/*set3*Δ | DHCA329 | **a**/**a** | DHCA226 | *set1*Δ::*C.d.HIS1*/*set1*Δ::*C.m.LEU2*  *SET3*/*set3*Δ::FRT | [5] |
| *set1*Δ/*set1*Δ *SET3-3HA*/*set3*Δ | DHCA506 | **a**/**a** | DHCA329 | *set1*Δ::*C.d.HIS1*/*set1*Δ::*C.m.LEU2*  *SET3-3HA-NAT1*/*set3*Δ::FRT | This study |
| *set1*Δ/*set1*Δ *SET3-9myc*/*set3*Δ | DHCA507 | **a**/α | SN152 | *set1*Δ::*C.d.HIS1*/*set1*Δ::*NAT1*  *SET3-9myc-C.m.LEU2*/*set3*Δ::FRT | This study |
| *BRG1*/*brg1*Δ | DHCA508 | **a**/α | CAIF-100 | *BRG1*/*brg1::C.d.ARG4* | This study |
| *brg1*Δ/*brg1*Δ | DHCA509 | **a**/α | CAIF-100 | *brg1::C.d.ARG4*/*brg1::NAT1* | This study |
| *TEC1*/*tec1*Δ | DHCA510 | **a**/α | CAIF-100 | *TEC1*/*tec1::C.d.ARG4* | This study |
| *TEC1*/*tec1*Δ *BRG1*/*brg1*Δ | DHCA511 | **a**/α | CAIF-100 | *TEC1*/*tec1::C.d.ARG4*  *BRG1*/*brg1::NAT1* | This study |
| *set3*Δ/*set3*Δ *BRG1*/*brg1*Δ | DHCA512 | **a**/α | SN152 | *set3*Δ::*C.d.HIS1*/*set3*Δ::*C.m.LEU2*  *BRG1*/*brg1::C.d.ARG4* | This study |
| *set3*Δ/*set3*Δ *brg1*Δ/*brg1*Δ | DHCA513 | **a**/α | SN152 | *set3*Δ::*C.d.HIS1*/*set3*Δ::*C.m.LEU2*  *brg1::C.d.ARG4*/*brg1::NAT1* | This study |
| *set3*Δ/*set3*Δ *TEC1*/*tec1*Δ | DHCA514 | **a**/α | SN152 | *set3*Δ::*C.d.HIS1*/*set3*Δ::*C.m.LEU2*  *TEC1*/*tec1::C.d.ARG4* | This study |
| *set3*Δ/*set3*Δ  *TEC1*/*tec1*Δ *BRG1*/*brg1*Δ | DHCA515 | **a**/α | SN152 | *set3*Δ::*C.d.HIS1*/*set3*Δ::*C.m.LEU2*  *TEC1*/*tec1::C.d.ARG4*  *BRG1*/*brg1::NAT1* | This study |
| *SET3-9myc*/*set3*Δ *NRG1-3HA*/*nrg1*Δ | DHCA516 | **a**/α | DHCA502 | *NRG1-3HA-NAT1*/*nrg1*Δ::FRT | This study |
| Brg1-myc | CJN1734 | **a**/α |  | *BRG1-13XMyc-FRT*/*brg1*Δ | [6] |
| Efg1-myc | CJN1781 | **a**/α |  | *EFG1-13XMyc-FRT*/*efg1*Δ | [6] |
| *tec1*Δ/*tec1*Δ | CJN2320 | **a**/α |  | *tec1*Δ::*C.d.HIS1*/*tec1*Δ::*C.m.LEU2* | [6] |

**SUPPLEMENTAL REFERENCES**

1. Gillum AM, Tsay EY, Kirsch DR (1984) Isolation of the *Candida albicans* gene for orotidine-5'-phosphate decarboxylase by complementation of *S. cerevisiae* *ura3* and *E. coli* *pyrF* mutations. Mol Gen Genet 198: 179-182.

2. Noble SM, Johnson AD (2005) Strains and strategies for large-scale gene deletion studies of the diploid human fungal pathogen *Candida albicans*. Eukaryot Cell 4: 298-309.

3. Frohner IE, Bourgeois C, Yatsyk K, Majer O, Kuchler K (2009) *Candida albicans* cell surface superoxide dismutases degrade host-derived reactive oxygen species to escape innate immune surveillance. Mol Microbiol 71: 240-252.

4. Hnisz D, Majer O, Frohner IE, Komnenovic V, Kuchler K (2010) The Set3/Hos2 histone deacetylase complex attenuates cAMP/PKA signaling to regulate morphogenesis and virulence of *Candida albicans*. PLoS Pathog 6: e1000889.

5. Hnisz D, Schwarzmuller T, Kuchler K (2009) Transcriptional loops meet chromatin: a dual-layer network controls white-opaque switching in *Candida albicans*. Mol Microbiol 74: 1-15.

6. Nobile CJ, Fox EP, Nett JE, Sorrells TR, Mitrovich QM, et al. (2012) A recently evolved transcriptional network controls biofilm development in *Candida albicans*. Cell 148: 126-138.
