# Supplementary material for: A Histone Deacetylase Adjusts Transcription Kinetics at Coding Sequences during Candida albicans Morphogenesis
Source: PLoS Genet. 2012 Dec 6;8(12):e1003118. doi: 10.1371/journal.pgen.1003118 (PMC3516536; doi:10.1371/journal.pgen.1003118)
Supplement: Table S2 — List of primers used in the study. (DOC) [file pgen.1003118.s009.doc]

**Table S2.** **Oligonucleotide primers used in this study**

| **Gene deletion constructs based on fusion PCR strategy** | | |
| --- | --- | --- |
| **Name** | **Description** | **Sequence (5´- 3´)*** |
| 55_BRG1 | BRG1 5´ flank | TGCAGCTTTTGTACTACATTTGG |
| 53_BRG1 | BRG1 5´ flank | cacggcgcgcctagcagcggAATTTGAATTTCTGGAATAGTAGTG |
| 35_BRG1 | BRG1 3´ flank | gtcagcggccgcatccctgcATTGCCTCCTTTGATGCACG |
| 33_BRG1 | BRG1 3´ flank | TCGGTTTGATCCAGCTCAGG |
| 55_TEC1 | TEC1 5´ flank | TAGACAGGGACCTTGACTTCCC |
| 53_TEC1 | TEC1 5´ flank | cacggcgcgcctagcagcggGTAGCACTAGGAGTAGCTTGCGAC |
| 35_TEC1 | TEC1 3´ flank | gtcagcggccgcatccctgcACTGTTGGATACATTAGTTTAGCG |
| 33_TEC1 | TEC1 3´ flank | AATGCTCAATAACCTCCCACC |
| 55_CA6009 | SET1 5´ flank | CGGTTTTCACTTAACTGCTGC |
| 53_CA6009 | SET1 5´ flank | cacggcgcgcctagcagcggCATAGCAAGGTGTATGTTTGATTAC |
| 35_CA6009 | SET1 3´ flank | gtcagcggccgcatccctgcTAATTGCATAAACGTGTGATAAATC |
| 33_CA6009 | SET1 3´ flank | TTAGTAGTAGGCGAATAGACACAGAC |
| M5 | Marker upstream | ccgctgctaggcgcgccgtgACCAGTGTGATGGATATCTGC |
| M3 | Marker downstream | gcagggatgcggccgctgacAGCTCGGATCCACTAGTAACG |
|  |  |  |
| **Gene tagging constructs based on fusion PCR strategy** | | |
| **Name** | **Description** | **Sequence (5´- 3´)** |
| 55_GFPSET3 | SET3 (3HA) 5´ flank | GCTGACGATAGTAAAGGTGAGAAG |
| 53_GFPSET3 | SET3 (3HA) 5´ flank | TTTCATTTTTTTATAATCAGCAAAAG |
| 55_GFPSET3 | SET3 (9myc) 5´ flank | GCTGACGATAGTAAAGGTGAGAAG |
| 53_9mycSET3 | SET3 (9myc) 5´ flank | CCACTAGCAGCAGAACCGGATTTCATTTTTTTATAATCAGCAAAAG |
| 35_GFPSET3 | SET3 tagging 3´ flank | TGCTGCAGGTCGACCGGTGTCGGGGCGACTTTGTAAACTTGATGAC |
| 33_GFPSET3 | SET3 tagging 3´ flank | CCAATGTACCACATCAAGGG |
| 55_3HAHOS2 | HOS2 (3HA) 5´ flank | CATTTGGAATACCAATGTTGG |
| 53_3HAHOS2 | HOS2 (3HA) 5´ flank | AGTCATTAGTTCTCCTAGTTTGG |
| 55_3HAHOS2 | HOS2 (9myc) 5´ flank | CATTTGGAATACCAATGTTGG |
| 53_9mycHOS2 | HOS2 (9myc) 5´ flank | CCACTAGCAGCAGAACCGGAAGTCATTAGTTCTCCTAGTTTGG |
| 55_3HAHOS2 | HOS2 (GFP) 5´ flank | CATTTGGAATACCAATGTTGG |
| 53_3HAHOS2 | HOS2 (GFP) 5´ flank | AGTCATTAGTTCTCCTAGTTTGG |
| 35_3HAHOS2 | HOS2 tagging 3´ flank | TGCTGCAGGTCGACCGGTGTCGGTCGTGGAGAAAGTCGAATCG |
| 33_3HAHOS2 | HOS2 tagging 3´ flank | GGGTACTGTCGTTGTATATGTATTCTC |
| 55_HANRG1 | NRG1 (3HA) 5´ flank | CCCAAGAAGAAAACACGTCTGC |
| 53_HANRG1 | NRG1 (3HA) 5´ flank | gatgttTACTAGGCTCTTGGTGTTGTATTTTG |
| 35_HANRG1 | NRG1 (3HA) 3´ flank | gtcagcggccgcatccctgcCTACATTGTTGTTCCATTTCTTTCG |
| 3C_HANRG1 | NRG1 (3HA) 3´ flank | AGGTGGATATGCTATCACTTGGTG |
| M5HA_NRG1 | 3HA-NAT1 for NRG1 | caaaatacaacaccaagagcctagtaAACATCTTTTACCCATACGATG |
| M3HA_NRG1 | 3HA-NAT1 for NRG1 | gcagggatgcggccgctgacGCAGGTTAACCTGGCTTATCG |
| M5_3HASET3 | 3HA-NAT1 for SET3 | CTTTTGCTGATTATAAAAAAATGAAAAACATCTTTTACCCATACGATG |
| M3_3HAHOS2 | 3HA-NAT1 for SET3 | CCGACACCGGTCGACCTGCAGCAGCAGGTTAACCTGGCTTATCG |
| M5_9mycSET3 | 9myc-LEU2 for SET3 | CTTTTGCTGATTATAAAAAAATGAAATCCGGTTCTGCTGCTAGTGG |
| M3N1_9mycHOS2 | 9myc-LEU2 for SET3 | CCGACACCGGTCGACCTGCAGCAGCCAGTGTGATGGATATCTGC |
| M5_3HAHOS2 | 3HA-NAT1 for HOS2 | CCAAACTAGGAGAACTAATGACTAACATCTTTTACCCATACGATG |
| M3_3HAHOS2 | 3HA-NAT1 for HOS2 | CCGACACCGGTCGACCTGCAGCAGCAGGTTAACCTGGCTTATCG |
| M5_9mycHOS2 | 9myc-LEU2 for HOS2 | CCAAACTAGGAGAACTAATGACTTCCGGTTCTGCTGCTAGTGG |
| M3N1_9mycHOS2 | 9myc-LEU2 for HOS2 | CCGACACCGGTCGACCTGCAGCAGCCAGTGTGATGGATATCTGC |
| M5_GFPHOS2 | GFP-NAT1 for HOS2 | CCAAACTAGGAGAACTAATGACTTCTAAAGGTGAAGAATTATTCAC |
| M3_3HAHOS2 | GFP-NAT1 for HOS2 | CCGACACCGGTCGACCTGCAGCAGCAGGTTAACCTGGCTTATCG |
|  |  |  |
| **Quantitative Real-time PCR** | | |
| **Name** | **Target locus** | **Sequence (5´- 3´)** |
| AHO294 | ADE2 | GTTGTCAGATCATTAGAAGGGGAAG |
| AHO295 | ADE2 | AAGTATCTGGGATCCTGGCA |
| RT50_PFK1 | PFK1 (probe 1) | TGTGATGGTTCCTCTCTCTCACAC |
| RT30_PFK1 | PFK1 (probe 1) | TTTTTGGAGATTATATTTCGGGG |
| RT51_PFK1 | PFK1 (probe 2) | TCGTCCTTGGCAAGAAATGG |
| RT31_PFK1 | PFK1 (probe 2) | GACTTCAAAGGGGTTTCGCC |
| RT54_PFK1 | PFK1 (probe 3) | CCAATTCGAGTTAAATCTGAGTTGG |
| RT34_PFK1 | PFK1 (probe 3) | TCAACTACCGATTCAAATTACTCGG |
| Tel7_2F1 | Chr 7 telomere | GGACTATTGGGGGTGCAAGC |
| Tel7_2R | Chr 7 telomere | GCCAATACAGGCCACACACC |
| RT5_3690inter | 19.3690 (probe 1) | GAGAGTGGAGAGTGGTGGAG |
| RT3_3690inter | 19.3690 (probe 1) | GAACTACAAATTAGGGGACGAAGG |
| RT5_3690.2 | 19.3690.2 (probe 2) | GGTGTAGTACTTCAGTCAATGG |
| RT3_3690.2 | 19.3690.2 (probe 2) | GGAGATAGAACATCAAACAACAACC |
| RT5_tRCCG_001 | tR(CCG)1 (probe 3) | AATTTGGTGGGTTCAAAAATGAGGG |
| RT3_tRCCG_002 | tR(CCG)1 (probe 3) | CGCAGTGGTTATGATTATGGTCTC |
| RT5_3691 | 19.3691 (probe 4) | GTTGGAGCAGCAGGAATTTCCG |
| RT3_3691 | 19.3691 (probe 4) | TTGAACTGGTCGATTACGAACCC |
| RT5_CaRIP1 | RIP1 | TGCTGACAGAGTCAAGAAACC |
| RT3_CaRIP1 | RIP1 | GAACCAACCACCGAAATCAC |
| RT5_19.542 | HXK2 | GGTCATGGATTTCCCAACCG |
| RT3_19.542 | HXK2 | TCTCATGTGAGCTGGCAAAGC |
| RT5_19.903 | GPM1 | CGTCAGATTATCCGAAACTGGTC |
| RT3_19.903 | GPM1 | ATCCAAGGCAATGTTGGCAG |
| RT5_ENO1 | ENO1 | AACAAATCCAAATTGGGTGCC |
| RT3_ENO1 | ENO1 | TTGGCATTGGAAATGTTGGC |
| RT5_3651 | PGK1 | CAGCCTTGGTGACTTCTGGAC |
| RT3_3651 | PGK1 | GCTTCCCACTTGGGTAGACC |
| RT5_6814 | TDH3 | CACTGCTCCATCTGCTGATGC |
| RT3_6814 | TDH3 | GCAGCACCAGTGGAAGATGG |
| RT5_CaH4 | H4 | GAGGTGGTGTTAAACGTATTTCTG |
| RT3_CaH4 | H4 | CAACATCCAATGAAGTGACGG |
| RT5_19.4980 | HSP70 | ATCAAGCTGCTATGAACCCAGC |
| RT3_19.4980 | HSP70 | CAACTTGAATCACTGGTTTACCTGC |
| RT5_19.7502 | orf19.7502 | CAGGCTGTTAAGCCACAACAAC |
| RT3_19.7502 | orf19.7502 | TGACATTGACAAAATCAATCAAAGG |
| RT5_19.7150 | NRG1 | GGTTGCACGTTGTCGAAACC |
| RT3_19.7150 | NRG1 | TGTTGCTGCTGCTGCTTGG |
| RT5_19.4056 | BRG1 | ACGATCAACCATTAGTGGAGG |
| RT3_19.4056 | BRG1 | GAAGAAGTAGGTGTAGATGATCCAC |
| RT5_TEC1 | TEC1 | TGAGCAACAACAACAACAACCAC |
| RT3_TEC1 | TEC1 | CTGGGTTGTTGTCATAGTGGCC |
| RT5_EFG1 | EFG1 | CATCACAACCAGGTTCTACAACCAAT |
| RT3_EFG1 | EFG1 | CTACTATTAGCAGCACCACCC |
| RT5_TYE7 | TYE7 | GGACTATTGGCTAACGATATGTCG |
| RT3_TYE7 | TYE7 | GCAAATTCTGTTCCGATCTCGC |
| RT5_NDT80 | NDT80 | CTCAACAAGGCCCAACACCTC |
| RT3_NDT80 | NDT80 | TTGACGTGGTTGTCTTGCTGG |
| RT5_CPH2 | CPH2 | AAGTCGTCCATTACATGAAGTCG |
| RT3_CPH2 | CPH2 | AGTCACTATGGAGTCAACGGAG |
| RT5_SSN6 | SSN6 | AACAACAGCAGCAACAACAGCG |
| RT3_SSN6 | SSN6 | CTGTCGCACGTTCAATGTCACC |
| RT5_CUP9 | CUP9 | GGTAATGCTCCATTGCTTCCTC |
| RT3_CUP9 | CUP9 | TGTTGCTGTGGAGGTGGTGG |
| RT5_ROB1 | ROB1 | CTGGATTCATCTCTTGGTTCACC |
| RT3_ROB1 | ROB1 | TGTTGTTGGTTAAGGTGGACGTG |
| RT5_GCN4 | GCN4 | CAGCACCAATTTGGATGGAGTC |
| RT3_GCN4 | GCN4 | GTTCTTTGCTAGTAGCACCAGC |
| RT5_CaTUP1 | TUP1 | GATGGCGATAGGTTGGTTTCAG |
| RT3_CaTUP1 | TUP1 | ACAAAGTCAAGGAACACTGGGAG |
| RT5_RIM101 | RIM101 | CAATCTAACACCACAGCTCTGC |
| RT3_RIM101 | RIM101 | GGTAGCCATAAGTTGGTTGGTG |
| RT5_19.5760 | IHD1 | TCTGCTCAAGACCTCCGTGC |
| RT3_19.5760 | IHD1 | ATCCAAGGCAATGTTGGCAG |
| RT5_HGC1 | HGC1 | GTCAGCTTCCTGCACCTCATC |
| RT3_HGC1 | HGC1 | AAACAGCACGAGAACCAGCG |
| RT5_UME6 | UME6 | TCATTCAATCCTACTCGTCCACC |
| RT3_UME6 | UME6 | CCAGATCCAGTAGCAGTGCTG |
| RT5_CaHWP1 | HWP1 | GAAACCTCACCAATTGCTCCAG |
| RT3_CaHWP1 | HWP1 | GTAGAGACGACAGCACTAGATTCC |
| CJNO1396 | TEC1 promoter | TGACAAAAGAACTTGATTAAAACTAATATTGAACTAATAAATG |
| CJNO1397 | TEC1 promoter | TGTAATTAAGGTATTCCATGCGCTCA |
| CJNO1402 | EFG1 promoter | CTTACCTCGGATCTCACTCTCTATTTG |
| CJNO1403 | EFG1 promoter | CGTGTTTTTGTTTTCAGCACCTATACG |
| CJNO1406 | BRG1 promoter | AATCCGACATCGTTTCTCTACACC |
| CJNO1407 | BRG1 promoter | GAAATAAAATCACATAATCTAACAAAATATGGAAGAGC |
| CJNO1410 | NRG1 promoter | AGTCAATTATGTTGCACAAATCCCG |
| CJNO1411 | NRG1 promoter | ACTCTTCTTGAATCTAAACAAACCTATAAAACC |

* Lower case and upper case letters denote exogenous and endogenous sequences, respectively.
