# Supplementary material for: A Histone Deacetylase Adjusts Transcription Kinetics at Coding Sequences during Candida albicans Morphogenesis
Source: PLoS Genet. 2012 Dec 6;8(12):e1003118. doi: 10.1371/journal.pgen.1003118 (PMC3516536; doi:10.1371/journal.pgen.1003118)
Supplement: Table S3 — List of plasmids used in the study. (DOC) [file pgen.1003118.s010.doc]

**Table S3. Plasmids used in this study**

| **Name** | **Parent** | **Targeted locus** | **Fragment / Descriprion** | **Reference** |
| --- | --- | --- | --- | --- |
| pSFS2A | - | - | *NAT1* donor | [1] |
| pDH104 | pSFS2A | *SET3* | *SET3* 5´and 3´ flank for knockout | [2] |
| pSFS2a-NRG1urdr | pSFS2A | *NRG1* | *NRG1* 5´and 3´ flank for knockout | [3] |
| pSN40 | - | - | *C.m.LEU2* marker donor | [4] |
| pSN69 | - | - | *C.d.ARG4* marker donor | [4] |
| pDH133 | pSN40 | - | 9myc-LEU2 donor | This study |
| pFA6a | - | - |  | [5] |
| pFA6a-3HA-NAT1 | pFA6a | - | 3HA-NAT1 donor | [3] |
| pFA6a-GFP-NAT1 | pFA6a | - | GFP-NAT1 donor | [3] |

**SUPPLEMENTAL REFERENCES**

1. Reuss O, Vik A, Kolter R, Morschhauser J (2004) The SAT1 flipper, an optimized tool for gene disruption in *Candida albicans*. Gene 341: 119-127.

2. Hnisz D, Schwarzmuller T, Kuchler K (2009) Transcriptional loops meet chromatin: a dual-layer network controls white-opaque switching in *Candida albicans*. Mol Microbiol 74: 1-15.

3. Tscherner M, Kuchler K unpublished.

4. Noble SM, Johnson AD (2005) Strains and strategies for large-scale gene deletion studies of the diploid human fungal pathogen *Candida albicans*. Eukaryot Cell 4: 298-309.

5. Longtine MS, McKenzie A, 3rd, Demarini DJ, Shah NG, Wach A, et al. (1998) Additional modules for versatile and economical PCR-based gene deletion and modification in *Saccharomyces cerevisiae*. Yeast 14: 953-961.
